# Diversity of Lignicolous Freshwater Fungi from Yuanjiang River in Yunnan (China), with the Description of Four New Species

**DOI:** 10.3390/jof10120881

**Published:** 2024-12-18

**Authors:** Liang Zhang, Dan-Feng Bao, Hong-Wei Shen, Zong-Long Luo

**Affiliations:** 1 College of Agriculture and Biological Science, Dali University, Dali 671003, China; zhangliangfungi@163.com (L.Z.); hongweifungi@outlook.com (H.-W.S.); 2 Engineering and Research Center for Southwest Biopharmaceutical Resource of National Education Ministry of China, Guizhou University, Guiyang 550025, China; baodanfeng0922@gmail.com; 3 Center of Excellence in Fungal Research, Mae Fah Luang University, Chiang Rai 57100, Thailand; 4 School of Science, Mae Fah Luang University, Chiang Rai 57100, Thailand; 5 Cangshan Forest Ecosystem Observation and Research Station of Yunnan Province, Dali University, Dali 671003, China

**Keywords:** four new species, lignicolous freshwater fungi, morphology, phylogeny, taxonomy

## Abstract

Yuanjiang River (Red River) is one of the six major water systems in Yunnan Province, which originates from western Yunnan Province. This river system features numerous tributaries, complex terrain, and abundant natural resources. During the investigation on the diversity of lignicolous freshwater fungi in the Yuanjiang River, nine species were collected and identified, five belonging to *Dothideomycetes* and four to *Sordariomycetes*. Based on morphology and multigene phylogenetic analyses, four species, namely, *Aquadictyospora aquatica*, *Dictyosporium fluminicola*, *Myrmecridium submersum*, and *Neomyrmecridium fusiforme*, are described as new species. *Dictyocheirospora aquadulcis* is reported as a new national record, and *Myrmecridium hydei* is reported as a new habitat record. *Dictyocheirospora rotunda*, *Halobyssothecium aquifusiforme*, and *Pseudohalonectria lutea* were known earlier from freshwater habitats, but we described them in detail in this paper. This study contributes significantly to the understanding of the diversity of lignicolous freshwater fungi in southwestern China.

## 1. Introduction

Lignicolous freshwater fungi grow on submerged woody debris in various freshwater habitats, such as streams, ponds, lakes, swamps, dams, and tree hollows [1,2]. These fungi can decompose lignocellulose substrates and release energy and nutrients into the water environment, and they play an important role in the freshwater environment [3,4,5]. They represent a highly diverse group within the kingdom Fungi, which comprises over 150,000 reported species worldwide, with more than 90,000 being ascomycetes, most of which are terrestrial [6,7]. In contrast, only around 3900 species are from freshwater environments. It shows the significance of continued exploration and suggests that vast freshwater ascomycetes remain undiscovered [8,9]. Current research on lignicolous freshwater fungi is predominantly focused on those of Asia, particularly in countries such as China and Thailand [1,10,11,12,13,14,15,16,17,18].

Yunnan Province is one of the most biologically diverse regions in China with abundant freshwater environments. Since 1986, over 281 species of lignicolous freshwater fungi have been reported from Yunnan [15]. These fungi mainly belong to the classes *Dothideomycetes* and *Sordariomycetes* of the phylum *Ascomycota*, with a few species belonging to the classes *Eurotiomycetes* and *Leotiomycetes* [15]. The Yuanjiang River originates at the eastern foothills of the Ailao Mountain in western Yunnan, China. The Yuanjiang River (Red River) is one of the six major water systems in Yunnan, which is the only significant international river that originates from Yunnan Province [19,20]. Known as the “Red River” due to the abundance of red sandstone formations in its basin, which turn the water crimson [19,20]. The Yuanjiang River extends from tropical regions to temperate zones in the central plateau of Yunnan. This region experiences high average annual temperatures and abundant precipitation, contributing to a large watershed with abundant runoff [21]. The unique geographical location and complex terrain have given rise to diverse ecosystems providing a suitable growth environment for abundant freshwater fungal resources. Given these favorable conditions, the Yuanjiang River has become an important area for studying lignicolous freshwater fungi. Despite their ecological importance, research on the lignicolous freshwater fungi of the Yuanjiang River remains limited. To address this gap, we are conducting a systematic study of lignicolous freshwater fungi in the Yuanjiang River. As part of an ongoing systematic study of these fungi in the region, three new species, namely, *Distoseptispora suae*, *Distoseptispora xinpingensis*, and *Wongia suae*, have been described [17,18].

In this study, nine species from the families *Dictyosporiaceae*, *Lentitheciaceae*, *Myrmecridiaceae*, and *Pseudohalonectriaceae* were isolated and identified. Based on morphological characters and multigene phylogenetic analysis, we report the finding of four new species (*Aquadictyospora aquatica*, *Dictyosporium fluminicola*, *Myrmecridium submersum*, and *Neomyrmecridium fusiforme*), two new records (*Dictyocheirospora aquadulcis* and *Myrmecridium hydei*), and three recollections (*Dictyocheirospora rotunda*, *Halobyssothecium aquifusiforme*, and *Pseudohalonectria lutea*), which are described and illustrated. The results not only enrich the knowledge of the diversity of lignicolous freshwater fungi in China but also provide recommendations and support for their conservation and utilization. Furthermore, they offer a reference for better understanding of the species diversity and distribution patterns of lignicolous freshwater fungi in the Yuanjiang River.

## 2. Materials and Methods

### 2.1. Isolation and Morphology

Samples of submerged, decaying wood were collected from a freshwater stream in the Yuanjiang River in Yunnan Province and brought to the laboratory in plastic bags. The samples were incubated in plastic boxes lined with moistened tissue paper at room temperature for one week. Specimen observations and morphological studies were conducted following the protocols provided by Luo et al. [10] and Senanayake et al. [22].

Macromorphological characteristics of specimens were observed using an Optec SZ 760 stereo microscope (Chongqing Optec Instrument Co., Ltd., Chongqing, China). Temporarily prepared microscope slides were placed under a Nikon ECLIPSE NiU compound microscope for observation and micromorphological photography. The morphologies of colonies on native substrates were photographed with a Nikon SMZ1000 stereo microscope (Nikon Corporation, Tokyo, Japan). Single spore isolation was performed as follows: the tip of a sterile toothpick dipped in sterile water was used to capture the conidia of the target colony directly from the specimen; the conidia were then streaked on the surface of water agar (WA, Composition: Agar 20 g/L, Chloramphenicol 0.1 g/L) or potato dextrose agar (PDA, CM123, Composition: Potato infusion 5.0 g/L, Dextrose 20 g/L, Agar 20 g/L, Chloramphenicol 0.1 g/L, from Beijing Bridge Technology Co., Ltd., Beijing, China) and incubated at room temperature overnight. The single germinated conidia were transferred to fresh PDA plates and incubated at room temperature. A few of the remaining germinated spores in the media plate were separated along with agar by using a needle and transferred onto water-mounted glass slides for photographs to capture the germination position of the germ tubes [16]. The specimens were deposited in the Herbarium of Cryptogams Kunming Institute of Botany, Academia Sinica (KUN-HKAS), Kunming, China. Living cultures were deposited in the China General Microbiological Culture Collection Center (CGMCC), Beijing, China, and the Kunming Institute of Botany Culture Collection Center (KUNCC), Kunming, China. Fungal Names number (FN) of the new species was registered (https://nmdc.cn/fungalnames/, accessed on 25 August 2024). New species were established following the recommendations outlined by Chethana et al. [23].

### 2.2. DNA Extraction, PCR Amplification, and Sequencing

Fungal mycelium was scraped from the edges of the growing culture using a sterile scalpel and transferred to a 1.5 mL microcentrifuge tube using sterilized inoculum needles. The Trelief™ Plant Genomic DNA Kit (Beijing TsingKe Biotech Co., Ltd., Beijing, China) was used to extract DNA from the ground mycelium according to the manufacturer’s instructions. Four gene regions, namely, ITS, LSU, SSU, and *tef*1-α, were amplified using ITS5/ITS4, LR0R/LR5, NS1/NS4, and 983F/2218R [24,25,26]. The amplification was performed in a 25 μL reaction volume containing 9.5 μL deionized water, 12.5 μL 2× Taq PCR Master Mix with blue dye (Sangon Biotech, Shanghai, China), 1 μL of DNA template, and 1 μL of each primer (10 μM). The amplification conditions for ITS, LSU, SSU, and *tef*1-α were followed, as described by Luo et al. [10]. PCR amplification was confirmed on 1% agarose electrophoresis gels stained with ethidium bromide. The sequences were carried out at Tsingke Biological Engineering Technology and Services Company, Kunming, China.

### 2.3. Phylogenetic Analyses

A BLAST search was performed on sequences with high similarity indices to find the closest matches with taxa. The sequences were aligned using the MAFFT online service: multiple alignment program MAFFT version 7.0 (http://mafft.cbrc.jp/alignment/server/index.html, accessed on 21 October 2024), and all parameters were set by default [27,28]. Aligned sequences of each gene region (ITS, LSU, SSU, and *tef*1-α) were combined and manually improved using BioEdit v.7.0.5.3 [29]. Ambiguous regions were excluded from the analysis, and gaps were treated as missing data.

Maximum likelihood analysis was performed at the CIPRES Science Gateway v.3.3 [30] using RAxML v. 8.2.8 as part of the “RAxML-HPC2 on XSEDE” tool [31,32]. All model parameters were estimated by RAxML. The final RAxML search was conducted using the GTRGAMMA+I model, which was estimated by using MrModeltest 2.2 [33]. Maximum likelihood bootstrap support was calculated from 1000 bootstrap replicates.

Bayesian analysis was performed using MrBayes v 3.1.2. [34]. The model of each gene was estimated using MrModeltest 2.2 [33]; the GTR + I + G model was the best-fit model of ITS, LSU, SSU, and *tef*1-α for Bayesian analysis. Posterior probabilities (PPs) [35] were performed by Markov chain Monte Carlo sampling (MCMC) in MrBayes v.3.1.2 [34]. Six simultaneous Markov chains were run for 10 million generations, and trees were sampled every 100th generation (resulting in 100,000 trees). The first 20,000 trees representing the burn-in phase of the analyses were discarded, and the remaining 80,000 (postburning) trees were used for calculating posterior probabilities (PPs) in the majority rule consensus tree [36,37]. Phylogenetic trees were represented by FigTree v 1.4.4 [38] and edited in Microsoft Office PowerPoint 2016. Sequences generated in this study were deposited in GenBank and are listed in Table S1.

## 3. Results

### 3.1. Taxonomy

#### 3.1.1. *Dothideomycetes* O.E. Erikss & Wink

*Pleosporales* Luttr. ex M.E. Barr
*Dictyosporiaceae* Boonmee & K.D. Hyde

*Notes*: *Dictyosporiaceae* was introduced by Boonmee et al. [39] to accommodate a holomorphic group with cheiroid, digitate, or palmate, multi-septate, brown, and/or dictyosporous conidia or coelomycetous with ellipsoidal or cylindrical, hyaline or brown, mostly aseptate conidia. The sexual morph of this family is characterized by globose to subglobose, superficial, dark brown to black ascomata; bitunicate, fissitunicate asci; and septate, hyaline ascospores with or without sheath. Most known members of *Dictyosporiaceae* are widely distributed as saprobes on plant debris from aquatic and terrestrial environments in temperate, tropical, and subtropical regions [16,39,40,41,42,43,44,45,46,47,48,49,50,51].

*Aquadictyospora* Z.L. Luo, K.D. Hyde & H.Y. Su

*Notes*: Based on morphological and phylogenetic analyses, Li et al. established *Aquadictyospora* with *A. lignicola* as the type species [42]. The genus is characterized by sporodochia, superficial, circular or subglobose conidiomata, micronematous conidiophores with monoblastic conidiogenous cells, and uniformly medium brown dictyosporous conidia with a subglobose, hyaline cell at the basal end [42]. *Aquadictyospora* have been reported on submerged decayed wood in freshwater habitats, as well as from dead stems of Clematis sikkimensis in terrestrial habitats in China and Thailand [42,52].

**Figure 1:**
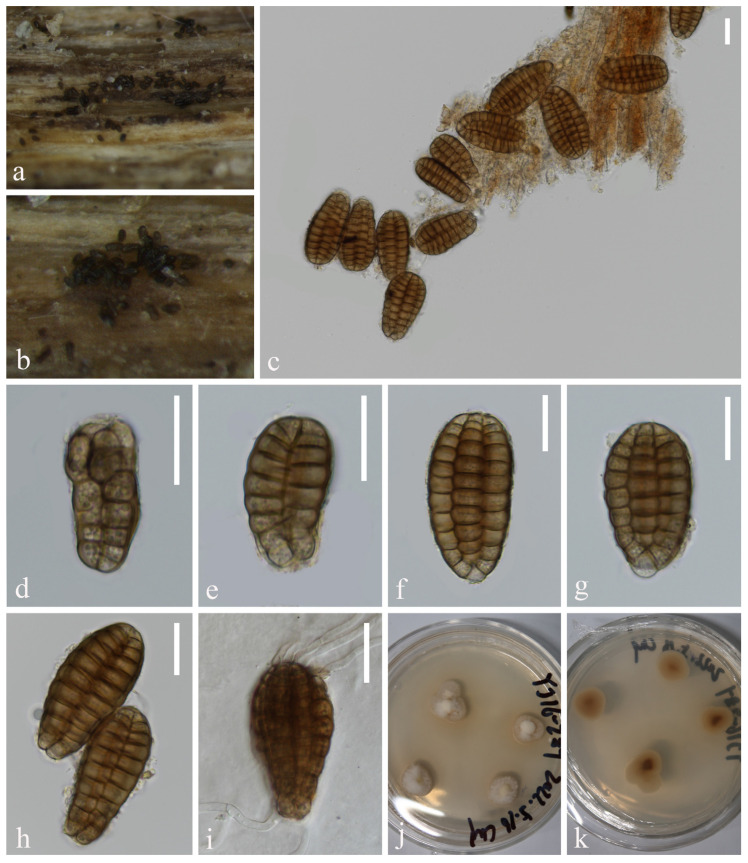
*Aquadictyospora aquatica* (KUN-HKAS 135199, holotype): (**a**,**b**) colonies on submerged decaying wood, (**c**–**h**) conidia, (**i**) germinating conidium, and (**j**,**k**) culture on PDA from surface and reverse. Scale bars: (**c**–**i**) 20 µm.

*Aquadictyospora aquatica* L. Zhang & Z.L. Luo, sp. nov., Figure 1

Fungal Names number: FN 572048

Etymology: Referring to the aquatic habitat of this fungus.

Holotype: KUN-HKAS 135199

*Saprobic* on submerged decaying wood in freshwater stream. **Sexual morph**: Undetermined. **Asexual morph**: Hyphomycetous. *Colonies* on natural substratum sporodochia, superficial, scattered, dark brown to black. *Mycelium* is immersed, composed of septate, branched, smooth, thin-walled hyphae. *Conidiophores are* micronematous, reduced to conidiogenous cells. *Conidiogenous cells* are holoblastic, hyaline to pale brown, mostly remaining attached to the conidia. *Conidia* 48–58 × 22–30 µm ($\bar{x}$ = 53 × 26 µm, n = 40), solitary, oval to ellipsoid, cheiroid, not complanate, brown, pale brown at base, consisting of 4–6 vertical rows of cells, with a basal connecting cell, slightly curved inward at the apex, hyaline or pale brown, each row composed of 5–11 cells, constricted at septa, guttulate, without appendages.

*Culture characteristics*: Conidia germinating on PDA within 12 h, and germ tubes are produced from both ends. Colonies on PDA reaching 23 mm in diameter after 4 weeks at 25 °C, in natural light, irregular, with fluffy, dense, pale brown mycelium on the surface, brown in reverse.

*Material examined*: China, Yunnan Province, on submerged decaying wood in a freshwater stream in the Yuanjiang River basin, 23°55′23″ N, 101°39′14″ E, 22 February 2022, Hong-Wei Shen, S-3657 (KUN-HKAS 135199, holotype), ex-type culture, KUNCC 23–17142.

**Figure 2:**
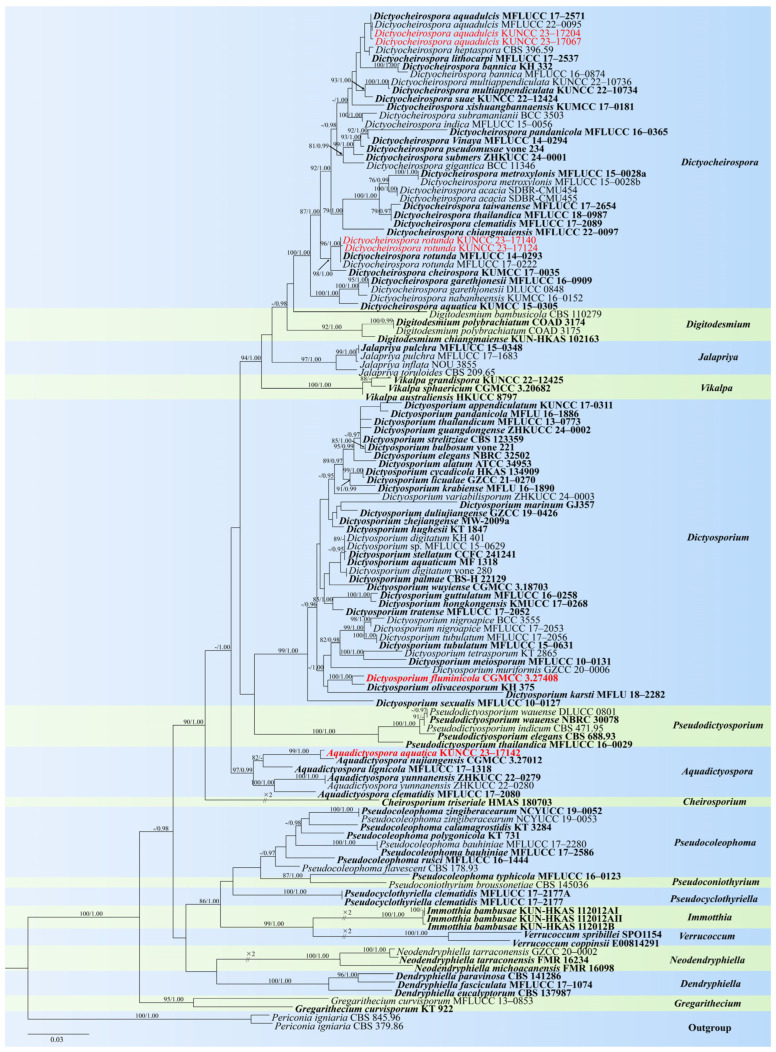
RAxML tree based on analysis of combined ITS, LSU, and *tef*1-α dataset. The combined analyses include 123 strains with 2665 characters, including gaps (ITS: 568 bp, LSU: 1255 bp, and *tef*1-α: 842 bp). The tree is rooted to *Periconia igniaria* (CBS 845.96 and CBS 379.86). The tree topology of the maximum likelihood analysis and Bayesian analysis are similar. The RAxML analysis of the combined dataset yielded a best-scoring tree with a final ML likelihood value of −22,329.430383. The matrix had 1134 distinct alignment patterns, with 33.49% undetermined characters or gaps. Estimated base frequencies were as follows: A = 0.239949, C = 0.251526, G = 0.270702, and T = 0.237823; substitution rates AC = 1.558395, AG = 3.722811, AT = 1.999671, CG = 0.838522, CT = 8.185164, and GT = 1.000000; and gamma distribution shape parameter α = 0.224379. Bootstrap values for maximum likelihood (ML) of ≥75% and clade credibility values of ≥0.95 from Bayesian-inference analysis are labeled on the nodes. The ex-type strains are in bold. The newly obtained sequences are indicated in red.

*Notes*: In our phylogenetic analysis, the new isolate *Aquadictyospora aquatica* (KUNCC 23–17142) clustered with *A. nujiangensis* (CGMCC 3.27012) with 100% ML and 1.00 PP support (Figure 2). A comparison of the ITS sequences between *A. aquatica* and *A. nujiangensis* showed 1.9% (10/522 bp, including gaps) nucleotide differences. Morphologically, *A. aquatica* resembles *A. nujiangensis* in having cheiroid, ellipsoid to cylindrical, and not complanate conidia. However, the conidiophores of *A. aquatica* are reduced to conidiogenous cells [53]. Therefore, *A. aquatica* is recognized as a phylogenetically distinct species and described in this study.

*Dictyocheirospora* D’souza, Boonmee & K.D. Hyde

*Notes*: *Dictyocheirospora* was introduced by Boonmee et al. [39] with *Di. rotunda* as the type species. *Dictyocheirospora* is characterized by cheiroid, non-complanate, cylindrical conidia with arms arising from the basal cell and mostly with conidial arms closely gathered together at the apex [39]. Twenty-nine species are accepted in the genus, including eight species transferred from *Dictyosporium*. *Dictyocheirospora* species were distributed worldwide, and most taxa were reported in freshwater and terrestrial habitats from China and Thailand [2,16,39,42,43,44,45,51,52,54,55,56,57,58,59,60]. Most members of the *Dictyocheirospora* species have sequence data and are grouped within a well-supported monophyletic clade [2,16,51,52,60].

*Dictyocheirospora aquadulcis* Sorvongxay, Boonmee & K.D. Hyde, Fungal Diversity 96: 23 (2019), Figure 3 and Figure 4

**Figure 3:**
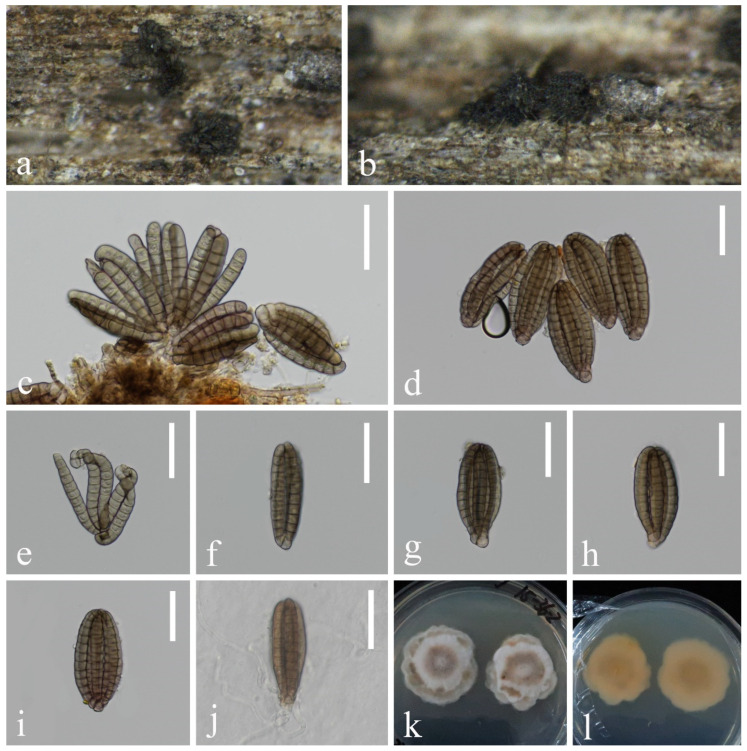
*Dictyocheirospora aquadulcis* (KUN-HKAS 132119, new national record): (**a**,**b**) colonies on submerged decaying wood, (**c**) conidiomata and conidia, (**d**–**i**) conidia, (**j**) germinating conidium, and (**k**,**l**) culture on PDA from surface and reverse. Scale bars: (**c**–**j**) 20 µm.

**Figure 4:**
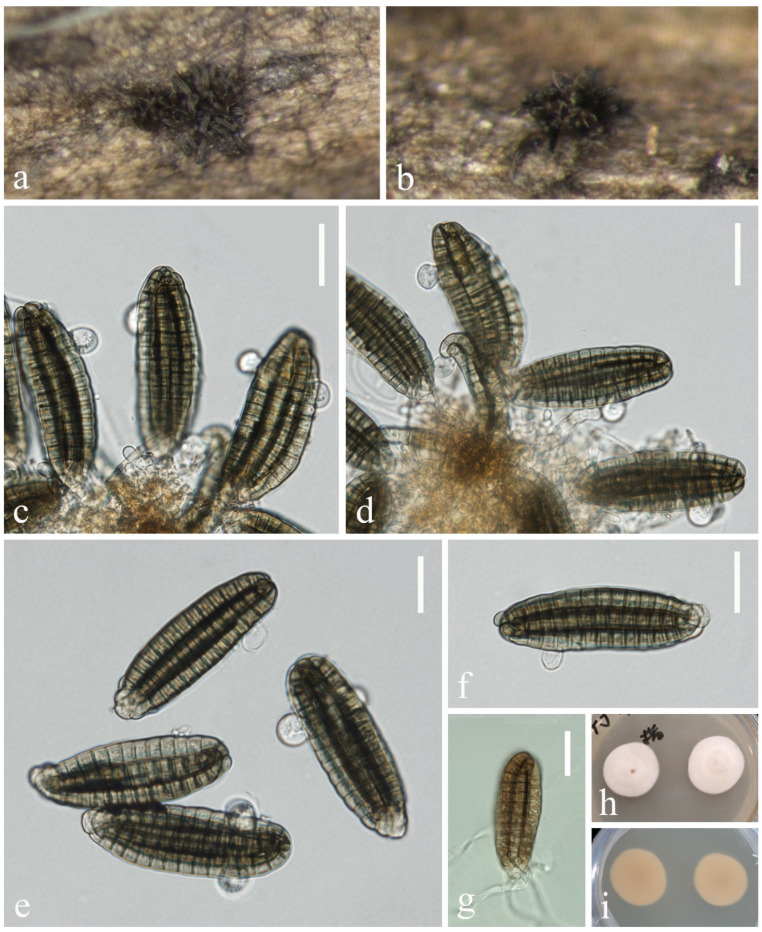
*Dictyocheirospora aquadulcis* (KUN-HKAS 132124): (**a**,**b**) colonies on submerged decaying wood, (**c**,**d**) conidiomata and conidia, (**e**,**f**) conidia with appendages, (**g**) germinating conidium, and (**h**,**i**) culture on PDA from surface and reverse. Scale bars: (**c**–**g**) 20 µm.

Index Fungorum number: IF556308

*Saprobic* on submerged decaying wood in freshwater stream. **Sexual morph**: Undetermined. **Asexual morph**: Hyphomycetous. *Colonies* on natural substrate, sporodochial, punctiform, velvety, superficial, scattered, and dark brown to black. *Conidiophores* are micronematous, pale brown, smooth, thin-walled, and sometimes reduced to conidiogenous cells. *Conidiogenous cells* are holoblastic, cylindrical, sometimes flat at the base, hyaline to pale brown, mostly remaining attached to the conidia. *Conidia* 49–63 × 17–27 µm ($\bar{x}$ = 56 × 22 µm, n = 25), solitary, oval to ellipsoid, cheiroid, not complanate, brown, consisting of 5–7 vertical rows of cells, with a basal connecting cell, slightly curved inward at the apex, separated when immersed in water, each row composed of 5–12 cells, constricted at septa, guttulate, without appendages (KUN-HKAS 132119, Figure 3). *Conidia* 63–70 × 20–23 µm ($\bar{x}$ = 67 × 22 µm, n = 30), solitary, cheiroid, brown, not complanate, consisting of 5–7 vertical rows of cells, with a basal connecting cell, separated when immersed in water, each row composed of 12–15 cells, constricted at septa and with 1–2 hyaline, globose to subglobose subapical appendages (KUN-HKAS 132124, Figure 4).

*Culture characteristics*: Conidia germinating on PDA within 24 h, and germ tubes are produced from the basal cell. Colonies on PDA reaching 17–23 mm in diameter in 2 weeks at 25 °C, in natural light, circular, irregular, divide into two layers on the surface, with fluffy, dense, brown to white mycelium in the center layer and with a brown to white margin, raised on surface media, in reverse brown in the middle and pale brown at the margin (KUN-HKAS 132119, Figure 3). Colonies on MEA reaching 5–10 mm in diameter in a week at 25 °C, in natural light, circular, with fluffy, dense, white mycelium on the surface with an entire margin, pale brown in reverse (KUN-HKAS 132124, Figure 4).

*Material examined*: CHINA, Yunnan Province, on submerged decaying wood in a freshwater stream in the Yuanjiang River basin, 24°0′47″ N, 101°37′16″ E, 22 February 2022, Hong-Wei Shen, H-809 (KUN-HKAS 132119), living cultures, KUNCC 23–17067; ibid. on submerged decaying wood in a freshwater stream in the Yuanjiang River basin, 23°38′30″ N, 101°54′32″ E, 22 February 2022, Hong-Wei Shen, S-3740 (KUN-HKAS 132124), living culture, KUNCC 23–17204.

*Notes*: In our phylogenetic analysis, the two new isolates (KUNCC 23–17067 and KUNCC 23–17204) clustered with *Dictyocheirospora aquadulcis* (Figure 2). There are only 2 bp nucleotide differences in the ITS sequence between our two new isolates (KUNCC 23–17067 and KUNCC 23–17204), and there were 3 bp nucleotide differences in the ITS sequence between the new isolates and the holotype (MFLUCC 17–2571). Morphologically, KUNCC 23–17067 resembles KUNCC 23–17204 in having solitary and cheiroid conidia. However, KUNCC 23–17204 has longer conidia (63–70 µm vs. 49–63 µm) and more cells in each row (12–15 vs. 5–12). Additionally, KUNCC 23-17204 has appendages, which are absent in KUNCC 23–17067. Environmental factors can significantly influence morphological variations within the same species, especially in fungi, leading to variations when grown under different conditions or on diverse substrates. This phenomenon is well-documented in mycology. Therefore, further research is necessary to explore these morphological and genetic differences in more detail to determine whether they should be classified as the same species or different species. Based on the morphological and phylogenetic analysis, we therefore identified our two new isolates as the same species. The morphology of our new isolates fits well with *D. aquadulcis*, except the new isolate (KUNCC 23–17067) has smaller conidia (49–63 µm vs. 60–80 µm). The holotype (MFLUCC 17–2571) lacks appendages [56], while our new isolate (KUNCC 23–17204) has hyaline, globose to subglobose appendages consistent with Shen’s study [16]. We, therefore, identified them as *D. aquadulcis* based on phylogenetic analysis and morphological evidence. The previously reported that *D. aquadulcis* was collected on submerged wood in freshwater from Thailand [56], while our two new collections were collected from China, which is the first report of this species in China.

*Dictyocheirospora rotunda* D’souza, Bhat & K.D. Hyde, Fungal Diversity 80: 465 (2016), Figure 5

**Figure 5:**
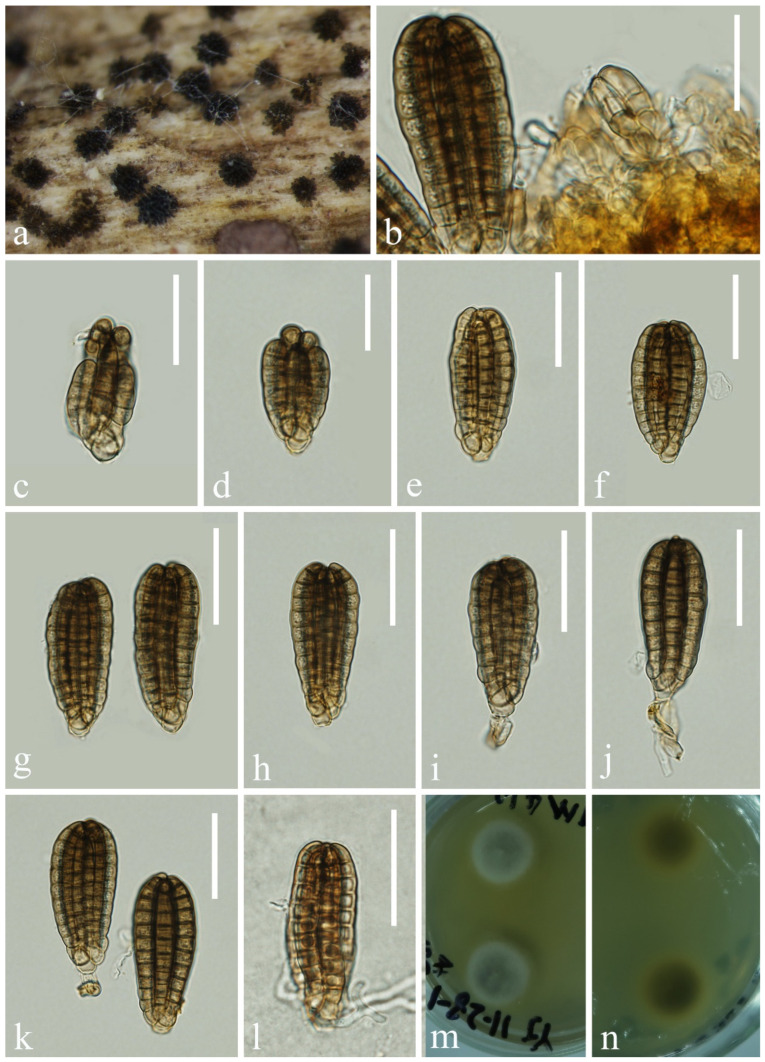
*Dictyocheirospora rotunda* (KUN-HKAS 132123): (**a**) colonies on submerged decaying wood, (**b**) conidiogenous cells with developing conidia, (**c**–**h**) conidia, (**i**–**k**) conidiogenous cells with conidia, (**l**) germinating conidium, and (**m**,**n**) culture on PDA from surface and reverse. Scale bars: (**b**–**d**) 20 µm, and (**e**–**l**) 30 µm.

Index Fungorum number: IF 551581

*Saprobic* on submerged decayed wood in freshwater stream. **Sexual morph**: Undetermined. **Asexual morph**: Hyphomycetous. *Colonies* on natural substrate forming sporodochial conidiomata, punctiform, velvety, superficial, scattered, and dark brown to black. Mycelium superficial, with brown, smooth, septate, branched hyphae. *Conidiophores* are micronematous, pale brown, and smooth-walled. *Conidiogenous cells* are holoblastic, integrated, terminal, cylindrical, sometimes flat at the base, hyaline to pale brown, mostly remaining attached to the conidia. *Conidia* 45–56 × 19–22 µm ($\bar{x}$ = 51 × 21 µm, n = 35), solitary, monoblastic, acrogenous, oval to ellipsoid, cheiroid, not complanate, brown, consisting of 5–8 vertical rows of cells, with a basal connecting cell, slightly curved inward at the apex, each row composed of 4–11 cells, constricted at septa, guttulate, with or without appendages.

*Culture characteristics*: Conidia germinating on PDA within 12 h, and germ tubes are produced from the basal cell. Colonies on PDA reaching 33 mm in diameter in 2 weeks at 25 °C, in natural light, are circular, with fluffy, dense, brown to white mycelium in the center, in reverse brown in the middle and pale brown at the margin.

*Material examined*: CHINA, Yunnan Province, on submerged decaying wood in a freshwater stream in the Yuanjiang River basin, 24°26′13″ N, 101°39′25″ E, 21 February 2022, Hong-Wei Shen, S-3459 (KUN-HKAS 132123), living cultures, KUNCC 23–17140; ibid. on submerged decaying wood in a freshwater stream in the Yuanjiang River basin, 25°28′05″ N, 100°09′39″ E, 19 February 2022, Hong-Wei Shen, S-3468 (KUN-HKAS 132126), living culture, KUNCC 23–17124.

*Notes*: The holotype of *Dictyocheirospora rotunda* was collected on submerged wood in freshwater habitat from Thailand [39]. In our phylogenetic analysis, the two new isolates (KUNCC 23–17140 and KUNCC 23–17124) clustered with *D. rotunda* (Figure 2). A comparison of ITS and LSU gene regions showed that the new isolates are almost identical to the ex-type strain of *D. rotunda* (MFLUCC 14–0293). The morphology of our new isolates fits well with *D. rotunda*, except conidia of our new isolates have hyaline appendages, which were not observed in *D. rotunda* [39]. Based on the morphological and phylogenetic analysis, we therefore identified our collections as *D. rotunda*. *D. rotunda* is widely dispersed in freshwater habitats in China and Thailand [2,39,42,43,44,50,52,54,55,56,57,58,61].

*Dictyosporium* Corda

*Notes*: *Dictyosporium* was established by Corda [62] with *D. elegans* as the type species; it is the holomorphic type genus of *Dictyosporiaceae* [39]. Asexual morphs of *Dictyosporium* are characterized by micronematous to macronematous, branched conidiophores, sometimes reduced to conidiogenous cells, solitary, cheiroid, digitate, and complanate, with pale brown or brown conidia with or without appendages. Sexual morphs have globose to subglobose ascomata, cylindrical asci, and hyaline, fusiform, uniseptate ascospores with or without a sheath [39,43,44,63]. Members of *Dictyosporium* species are mostly saprobes on plant litter in freshwater, marine, and terrestrial habitats [43,44,63,64,65,66,67,68].

**Figure 6:**
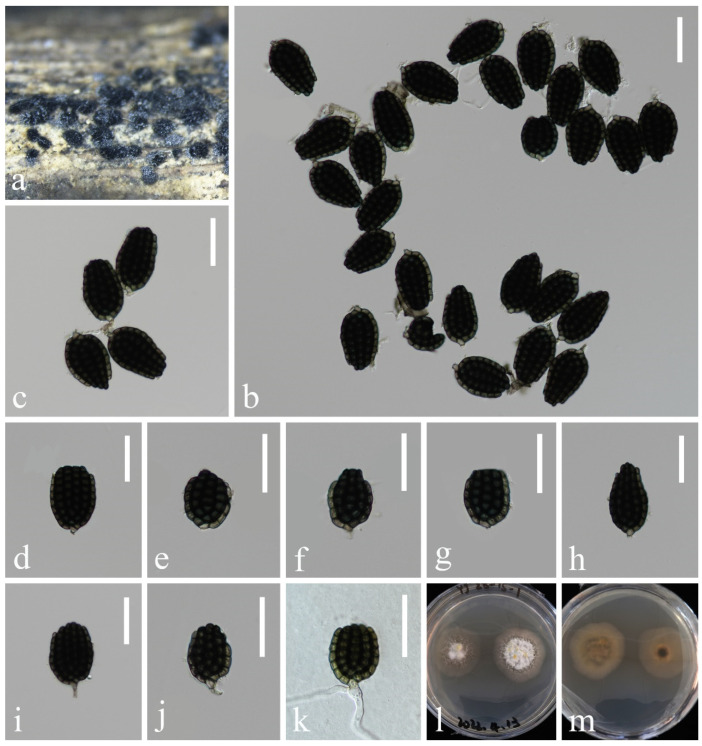
*Dictyosporium fluminicola* (KUN-HKAS 132125, holotype): (**a**) colonies on submerged decaying wood, (**b**) conidia heap, (**c**) conidiophores and conidia, (**d**–**h**) conidia, (**i**,**j**) conidiogenous cells and conidia, (**k**) germinating conidium, and (**l**,**m**) culture on PDA from surface and reverse. Scale bars: (**b**–**k**) 30 µm.

*Dictyosporium fluminicola* L. Zhang & Z.L. Luo, sp. nov., Figure 6

Fungal Names number: FN 572049

Etymology: In reference to the fungus dwelling in a stream.

Holotype: KUN-HKAS 132125

*Saprobic* on submerged decayed wood in freshwater stream. **Sexual morph**: Undetermined. **Asexual morph**: Hyphomycetous. *Colonies* on a natural substrate, punctiform, sporodochial, scattered, and dark brown to black. *Mycelium* mostly immersed, composed of smooth, hyaline to pale brown hyphae. *Conidiophores* are micronematous, hyaline or pale brown, smooth, and thin-walled. *Conidiogenous cells* are holoblastic, hyaline or pale brown, cylindrical, and smooth-walled. *Conidia* 33–45 × 22–27 µm ($\bar{x}$ = 39 × 25 µm, n = 45), solitary, ellipsoid to cylindrical, cheiroid, and complanate, composed of 5–6 arms close together, 4–9 euseptate in each arm, constricted at septa, dark olivaceous to brown, without appendages.

*Culture characteristics*: Conidia germinating on PDA within 24 h, and germ tubes are produced from the basal cell. Colonies on PDA reaching 46 mm in diameter in 2 weeks at 25 °C, in natural light, are circular, with flocculent, white mycelium in the center and with pale brown at the margin, in reverse brown spots in the middle and pale brown at the margin.

*Material examined*: CHINA, Yunnan Province, on submerged decaying wood in a freshwater stream in the Yuanjiang River basin, 23°03′45″ N, 103°16′07″ E, 24 February 2022, Hong-Wei Shen, S-3551 (KUN-HKAS 132125, holotype), ex-type culture, CGMCC 3.27408 = KUNCC 23–17212.

*Notes*: Phylogenetic analysis showed that *Dictyosporium fluminicola* (KUNCC 23–17212) clustered as a sister taxon to *Di. olivaceosporum* (KH 375) with 100% ML and 1.00 PP support (Figure 2). Morphologically, *Di. fluminicola* resembles *Di. olivaceosporum* in having cheiroid, ellipsoid to cylindrical conidia. However, *Di. olivaceosporum* (KH 375) has hyaline, globose to subglobose conidial appendages, which was distinct from our new species [39]. *Di. olivaceosporum* (KH 375) was collected from the submerged twigs in a stream in Japan, while our new species was collected from submerged decaying wood in China. A comparison of the ITS sequences between *Di. fluminicola* and *Di. olivaceosporum* showed 3.3% (12/360 bp) nucleotide differences, which support our new collection as a new species. Therefore, *Di. fluminicola* is introduced as a new species and described in this study.

*Lentitheciaceae* Y. Zhang, C.L. Schoch, J. Fourn, Crous & K.D. Hyde

Notes: *Lentitheciaceae* was introduced by Zhang et al. [69] to accommodate those lentitheciaceous taxa that have narrow peridia, fusiform to broadly cylindrical pseudoparaphyses, hyaline ascospores with 1–3 transverse septa and containing refractive globules, surrounded by a mucilaginous sheath or extended appendage-like sheaths, and asexual morphs are stagonospora-like or dendrophomalike [70,71,72,73]. Currently, 18 genera are accepted in *Lentitheciaceae*, with more than 100 species reported. Most members of *Lentitheciaceae* have been found as saprobes in aquatic and terrestrial habitats [74,75,76,77].

*Halobyssothecium* Dayarathne, E.B.G. Jones & K.D. Hyde

Notes: *Halobyssothecium* was introduced by Dayarathne et al. [78], and it is characterized by immersed, semi-immersed, erumpent, or superficial, carbonaceous or coriaceous, papillate, ostiolate ascomata; short-pedicellate, 8-spored asci; and fusiform, straight- or slightly curved, yellowish-brown, 1- or 3-septate, verrucose- or smooth-walled ascospores. The asexual morphs of this genus are coelomycetous or hyphomycetous [78]. There are 13 species accepted in *Halobyssothecium*, of which five species are known as the asexual morphs and the other eight species are known as sexual morphs [2,45,73,79]. Members of *Halobyssothecium* have a worldwide distribution from aquatic and terrestrial habitats in China, France, Gotland, Saudi Arabia, Thailand, and the United Kingdom [2,45,73,78,79].

*Halobyssothecium aquifusiforme* J. Yang, J.K. Liu & K.D. Hyde, Fungal Diversity 119: 39 (2023), Figure 7

**Figure 7:**
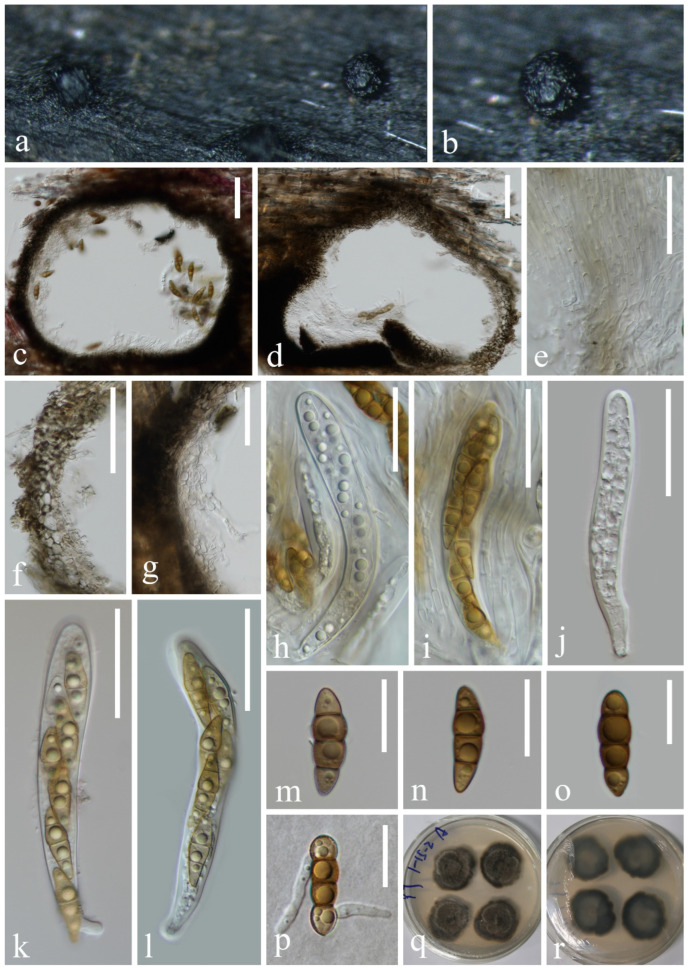
*Halobyssothecium aquifusiforme* (KUN-HKAS 132128): (**a**,**b**) appearance of ascomata on the host, (**c**,**d**) sections of ascomata, (**e**) pseudoparaphyses, (**f**,**g**) section of peridium, (**h**–**l**) asci, (**m**–**o**) ascospores, (**p**) germinated ascospore, and (**q**,**r**) colony on PDA from surface and reverse. Scale bar: (**c**,**d**) 60 µm, (**e**–**l**) 40 µm, and (**m**–**p**) 20 µm.

Index Fungorum number: IF559450

*Saprobic* on submerged decayed wood in freshwater stream. **Asexual morph**: Undetermined. **Sexual morph**: *Ascomata* 350–380 μm high, 320–370 μm in diameter, scattered or gregarious, immersed, perithecial, subglobose, dark brown to black. *Peridium* 16–33 µm thick, composed of several layers of pseudoparenchymatous cells, outer layers with brown, polygonal, thick-walled cells, inner layers with pale brown to hyaline, polygonal, thin-walled cells. *Pseudoparaphyses* 1.2–2.2 µm wide, branched, septate, and thickened at the septa, hyaline, filamentous, cylindrical. *Asci* 98–124 × 13–15 µm ($\bar{x}$ = 111 × 14 µm, n = 15), 8-spored, clavate to subcylindrical, bitunicate, fissitunicate, apex rounded, short pedicellate, with an ocular chamber. *Ascospores* 27–31 × 7–8 µm ($\bar{x}$ = 29 × 8 µm, n = 30), overlapping, uniseriate to biseriate, yellowish brown, 3-septate when mature, constricted at the septa, slightly curved, fusiform, guttulate, conical, and narrowly rounded at the ends; one cell on the central septum side is swollen, lacking gelatinous sheaths or appendages.

*Culture characteristics*: Ascospores germinating on PDA medium within 24 h, and germ tubes are produced from both ends. Colonies growing on PDA medium reaching 5–10 mm in 2 weeks at 25 °C in the dark, circular, raised, mycelium dense, velvety, gray-brown in the middle, olivaceous brown at the edge; from below, light brown at the center, dark brown at the margin.

*Material examined*: CHINA, Yunnan Province, on submerged decaying wood in a freshwater stream in the Yuanjiang River basin, 25°29′31″ N, 100°06′56″ E, 19 February 2022, Hong-Wei Shen, S-3430 (KUN-HKAS 132128), living cultures, KUNCC 23–17064.

**Figure 8:**
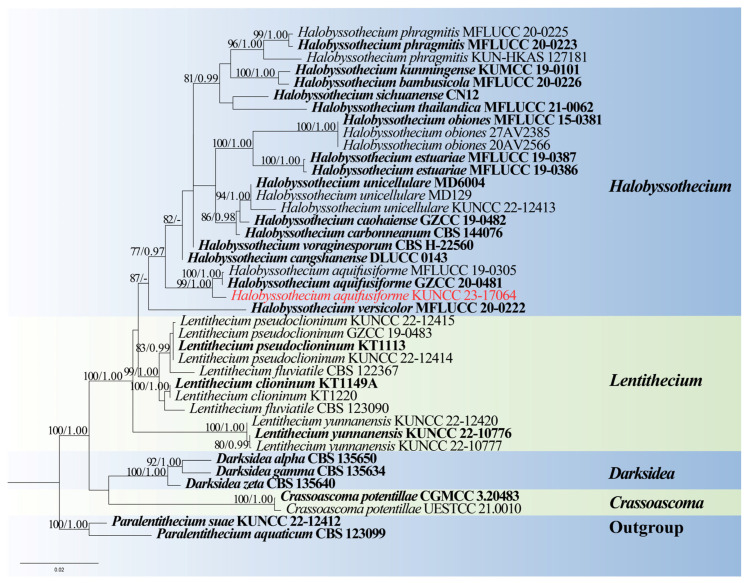
RAxML tree based on analysis of combined ITS, LSU, SSU, and *tef*1-α dataset. The combined analyses include 41 strains with 3416 characters, including gaps (ITS: 576 bp, LSU: 868 bp, SSU: 1020 bp, and *tef*1-α: 952 bp). The tree is rooted to *Paralentithecium aquaticum* (CBS 123099) and *Paralentithecium suae* (CGMCC 3.24265). The tree topology of the maximum likelihood analysis and Bayesian analysis are similar. The RAxML analysis of the combined dataset yielded a best-scoring tree with a final ML likelihood value of −10,581.198409. The matrix had 766 distinct alignment patterns, with 21.26% undetermined characters or gaps. Estimated base frequencies were as follows: A = 0.237748, C = 0.250655, G = 0.270916, and T = 0.240681; substitution rates AC = 0.899013, AG = 1.911393, AT = 0.973835, CG = 1.058482, CT = 5.445001, and GT = 1.000000; and gamma distribution shape parameter α = 0.020000. Bootstrap values for maximum likelihood (ML) ≥ 75% and clade credibility values ≥ 0.95 from Bayesian-inference analysis are labeled on the nodes. The ex-type strains are in bold. The newly obtained sequences are indicated in red.

*Notes*: Phylogenetic analysis showed that our new isolate clustered with the ex-type of *Halobyssothecium aquifusiforme* (GZCC 20–0481) with 99% ML and 1.00 PP support (Figure 8). Comparison of ITS, LSU, SSU, and *tef*1-α sequences between our new isolate and *H. aquifusiforme* (GZCC 20–0481) revealed 11 bp, 2 bp, 1 bp, and 9 bp differences, respectively, which were not significantly distinct from *H. aquifusiforme* (GZCC 20–0481). In addition, except for the size of asci (98–124 × 13–15 µm vs. 110–158 × 15–21 µm), our new collection is almost identical to the holotype of *H. aquifusiforme* in terms of immersed, globose conidiomata, yellowish brown, smooth, guttulate, and 3-septate ascospores [45]. We therefore identified our new collection as *H. aquifusiforme* and provide detailed descriptions and illustrations.

#### 3.1.2. *Sordariomycetes* O.E. Erikss. & Winka

*Myrmecridiales* Crous
*Myrmecridiaceae* Crous

*Notes*: *Myrmecridiaceae* is a monotypic family, which comprises two genera, including *Myrmecridium* and *Neomyrmecridium* [80,81], with *Myrmecridium* as the type genus [82]. Members of *Myrmecridiaceae* species are reported as saprobes on leaf litter, stems, or leaves of herbaceous plants from terrestrial and aquatic habitats worldwide, a few species occurring on soil and in house dust [81,82,83,84,85,86].

*Myrmecridium* Arzanlou, W. Gams & Crous

*Notes*: *Myrmecridium* was introduced by Arzanlou et al. [80], with *M. schulzeri* as the type species. *Myrmecridium* is characterized by its flat colonies, immersed mycelium that grows vertically and consists of unbranched, straight or flexuose, septate conidiophores. Conidiogenous cells are polyblastic, integrated, cylindrical, and solitary with obovoidal or fusiform, smooth, or finely verrucose-walled conidia [80]. Members of *Myrmecridium* have been found as saprobes in aquatic habitats from various substrates, such as submerged decaying wood, stems or leaves of herbaceous plants, dust, and soil [45,80,81,82,83,84,85,86,87,88,89,90,91,92,93]. Based on phylogenetic analysis and conidial characters, *M. aquaticum* and *M. sorbicola* were transferred to *Neomyrmecridium* [81,89]. So far, 25 species are accepted in *Myrmecridium* (Index Fungorum 2024, http://www.indexfungorum.org, accessed on 29 October 2024), and they are all known by their asexual morph, with the exception of *M. montsegurinum* [85].

*Myrmecridium hydei* Asghari, Phukhams & E.B.G. Jones, Phytotaxa 625: 265–279 (2023), Figure 9

**Figure 9:**
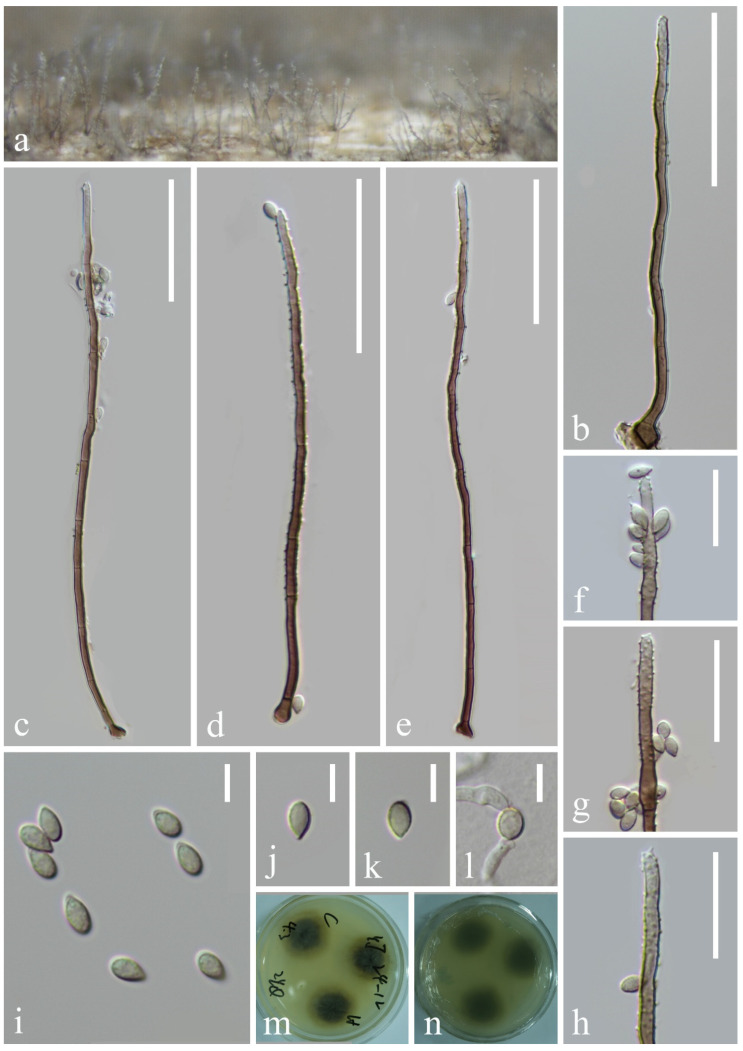
*Myrmecridium hydei* (KUN-HKAS 132120, new habitat record): (**a**) colonies on submerged decaying wood, (**b**–**e**) conidiophores and conidia, (**f**–**h**) conidiogenous cells, (**i**–**k**) conidia, (**l**) germinating conidium, and (**m**,**n**) culture on PDA from surface and reverse. Scale bars: (**b**–**e**) 40 µm, (**f**–**h**) 20 µm, and (**i**–**l**) 5 µm.

Index Fungorum number: IF 901177

*Saprobic* on submerged decayed wood in freshwater streams. **Sexual morph**: Undetermined. **Asexual morph**: Hyphomycetous. *Colonies* on natural substrate effuse, consisting of individual or fascicular conidiophores scattered over the substrate surface, are yellowish. *Mycelium* immersed and superficial, superficial hyphae pale brown, septate, cylindrical. *Conidiophores* 96–159 × 2–3 μm ($\bar{x}$ = 128 × 3 μm, n = 20), macronematous, mononematous, solitary or in small groups, erect, straight or slightly flexuous, subcylindrical, brown, paler towards apex, unbranched, septate with several denticles at apex. *Conidiogenous cells* polyblastic, integrated, terminal, subcylindrical, pale brown. *Conidia* 4–5 × 2–3 μm ($\bar{x}$ = 5 × 3 μm, n = 25), solitary, obovoid, tapering at the base, subhyaline to pale brown, aseptate, smooth, or finely verrucose-walled.

*Culture characteristics*: Conidia germinating on PDA within 12 h. Germ tubes are produced from both ends. Colonies on PDA reaching 44 mm in diameter after 2 weeks at 25 °C in natural light, with dense mycelium on the surface, brown in the middle, pale brown in the outer ring; in reverse, brown in the middle and paler at the entire margin.

*Material examined*: CHINA, Yunnan Province, on submerged decaying wood in a freshwater stream in the Yuanjiang River basin, 23°19′32″ N, 102°30′52″ E, 23 February 2022, Hong-Wei Shen, S-3387 (KUN-HKAS 132129), living cultures, KUNCC 23–17205; ibid. on submerged decaying wood in a freshwater stream in the Yuanjiang River basin, 23°12′04″ N, 102°54′28″ E, 24 February 2022, Hong-Wei Shen, S-3398 (KUN-HKAS 132120), living culture, KUNCC 23–17206.

**Figure 10:**
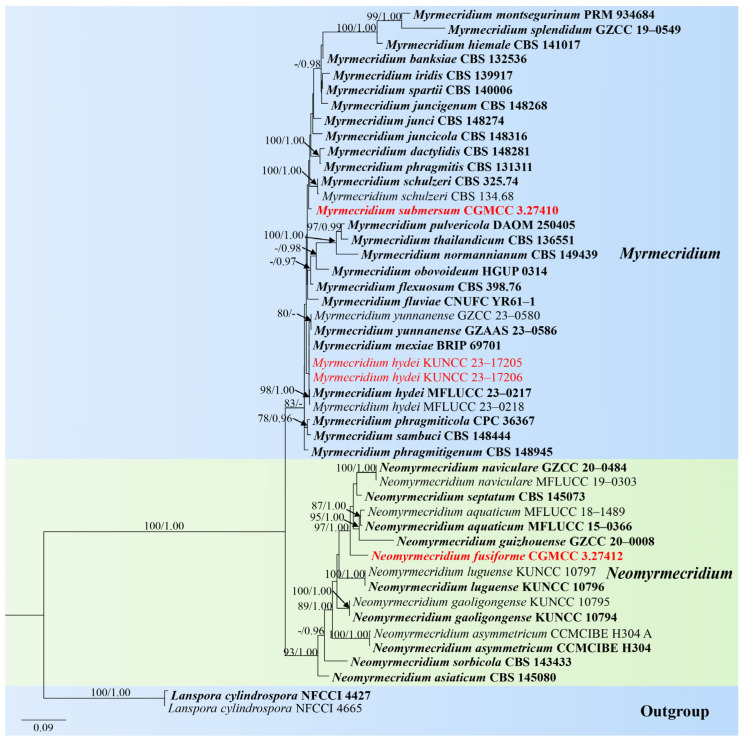
RAxML tree based on analysis of combined ITS and LSU dataset. The combined analyses include 47 strains with 1440 characters, including gaps (ITS: 556 bp and LSU: 884 bp). The tree is rooted to *Lanspora cylindrospora* (NFCCI 4427 and NFCCI 4665). The tree topology of the maximum likelihood analysis and Bayesian analysis are similar. The RAxML analysis of the combined dataset yielded a best-scoring tree with a final ML likelihood value of −7880.769866. The matrix had 543 distinct alignment patterns, with 8.41% undetermined characters or gaps. Estimated base frequencies were as follows: A = 0.245987, C = 0.247875, G = 0.286440, and T = 0.219698; substitution rates AC = 2.276747, AG = 2.342637, AT = 1.966718, CG = 0.662417, CT = 8.940493, and GT = 1.000000; and gamma distribution shape parameter α = 0.191988. Bootstrap values for maximum likelihood (ML) of ≥75% and clade credibility values of ≥0.95 from Bayesian-inference analysis are labeled on the nodes. The ex-type strains are in bold. The newly obtained sequences are indicated in red.

*Notes*: In the phylogenetic analysis, our strains (KUNCC 23–17205 and KUNCC 23–17206) clustered with *Myrmecridium hydei*, *M. mexiae*, and *M. yunnanense* [92,93,94] (Figure 10). Due to the lack of morphological information for *M. mexiae*, we only compare the morphology of *M. hydei*, *M. yunnanense*, and our strains. Morphologically, *M. yunnanense* (GZAAS 23–0586) resembled the holotype strain of *M. hydei* (MFLUCC 23–0217) in having macronematous, mononematous, unbranched, multi-septate conidiophores and aseptate, obovoid conidia, except for the size of conidiophores (70–105 × 4–5 µm in *M. yunnanense* vs. 115–250 × 2.5–4 µm in *M. hydei*) and conidiogenous cells (30–40 × 3.5–4 μm in *M. yunnanense* vs. 50–205 × 1.5–2.5 µm in *M. hydei*). Comparison of the ITS and LSU sequences of *M. yunnanense* (GZAAS 23–0586) and *M. hydei* (MFLUCC 23–0217) showed 99.41% (506/509 bp, including two gaps) and 99.24% (771/772 bp) sequence identity, respectively. Comparison of the ITS and LSU sequences of *M. mexiae* (BRIP 69701) and *M. hydei* (MFLUCC 23–0217) showed 99.43% (519/522 bp) and 99.88% (844/845 bp, including one gap) sequence identity, respectively. Based on morphological and phylogenetic analyses, we therefore identified that *M. hydei*, *M. mexiae*, and *M. yunnanense* were the same species. Because *M. hydei* was reported earlier, our new isolates were only compared with the type strain of *M. hydei* (MFLUCC 23–0217) in morphology and molecular characteristics. Furthermore, our isolates are morphologically fit with *M. hydei* (MFLUCC 23–0217); except for the size of conidiophores, our isolates have shorter conidiophores (96–159 vs. 115–250 µm). Comparison of ITS and LSU sequences between our new isolates and *M. hydei* (MFLUCC 23–0217) revealed 2 bp (including one gap) and 1 bp differences, respectively. Therefore, we identified the two new isolates as *M. hydei*. Our new collections were collected from freshwater habitats in China, while the holotype of *M. hydei* was collected from seawater in Thailand, *M. mexiae* was collected from leaves of *Sporobolus natalensis* (*Poaceae*) in Australia, and *M. yunnanense* was collected from leaves of *Cocos nucifera* in China. This is the first report of this species collected from freshwater habitat.

**Figure 11:**
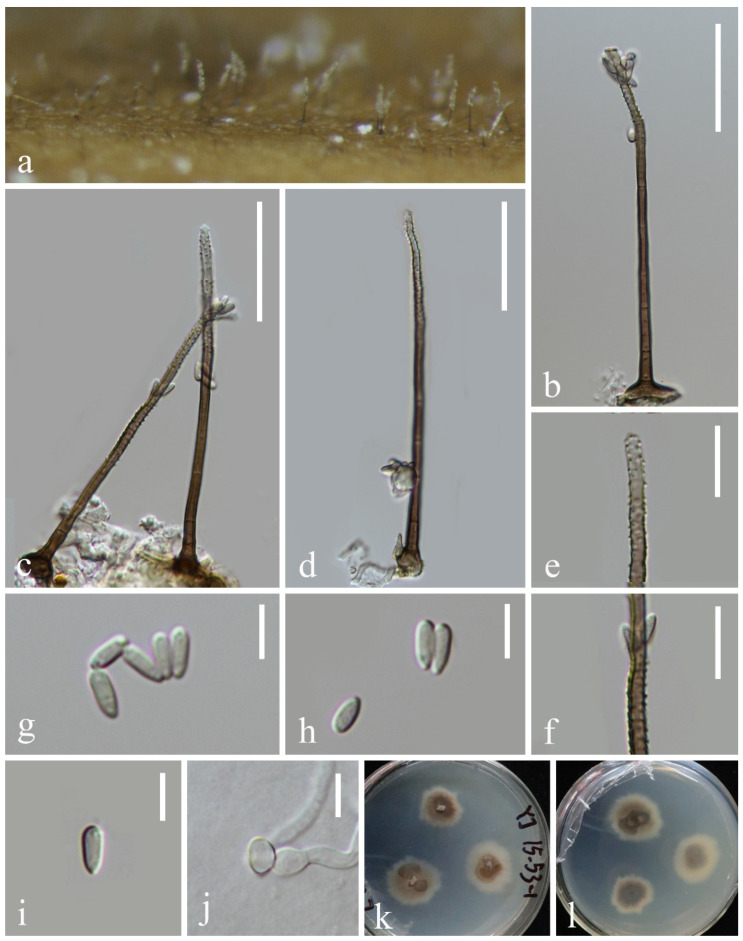
*Myrmecridium submersum* (KUN-HKAS 132127, holotype): (**a**) colonies on submerged decaying wood, (**b**–**d**) conidiophores and conidia, (**e**,**f**) conidiogenous cells, (**g**–**i**) conidia, (**j**) germinating conidium, and (**k**,**l**) culture on PDA from surface and reverse. Scale bars: (**b**–**d**) 30 µm, (**e**,**f**) 10 µm, and (**g**–**j**) 5 µm.

*Myrmecridium submersum* L. Zhang & Z.L. Luo, sp. nov., Figure 11

Fungal Names number: FN 572053

Etymology: Referring to the submerged habitats of the fungus.

Holotype: KUN-HKAS 132127

*Saprobic* on submerged decayed wood in freshwater streams. **Sexual morph**: Undetermined. **Asexual morph**: Hyphomycetous. *Colonies* on natural substrate effuse, greyish white, velvety. *Mycelium* immersed, composed of brown, branched, septate hyphae. *Conidiophores* 75–149 × 2–3 μm ($\bar{x}$ = 112 × 2 μm, n = 15), macronematous, mononematous, solitary, erect, straight or slightly flexuous, subcylindrical, unbranched, septate, paler towards apex, basal cell often inflated, smooth-walled with several denticles in the middle to upper part. *Conidiogenous cells* polyblastic, terminal, integrated, subcylindrical, subhyaline to pale brown, verrucose, with several denticles. *Conidia* 4–5 × 1–2 μm ($\bar{x}$ = 5 × 2 μm, n = 25), solitary, narrow, clavate, or subclavate, subhyaline, aseptate, smooth, or finely verrucose-walled.

*Culture characteristics*: Conidia germinating on PDA within 24 h. Germ tubes are produced from one or both ends. Colonies on PDA reaching 44 mm in diameter after 2 weeks at 25 °C in natural light, with dense mycelium on the surface, brown in the middle, subhyaline in the outer ring; in reverse, brown in the middle and subhyaline at the entire margin.

*Material examined*: China, Yunnan Province, on submerged decaying wood in a freshwater stream in the Yuanjiang River basin, 23°25′01″ N, 102°17′39″ E, 23 February 2022, Hong-Wei Shen, S-3335 (KUN-HKAS 132127, holotype), ex-type culture, CGMCC 3.27410 = KUNCC 23–17209.

*Notes*: Morphologies of *Myrmecridium* species are similar to each other, characterized by immersed mycelium that grows vertically and consists of unbranched, straight or flexuose, septate conidiophores, and conidiogenous cells are polyblastic, integrated, cylindrical with solitary, obovoidal or fusiform, smooth, or finely verrucose-walled conidia [80]. In our phylogenetic analyses, *Myrmecridium submersum* formed a distinct lineage within the genus and close to *M. schulzeri* [80] (Figure 10). They share some similar characteristics, such as unbranched, septate, conidiophores with inflated basal cells and solitary, subhyaline, aseptate, smooth, or finely verrucose-walled conidia [80]. However, conidia of *M. schulzeri* were surrounded by a wing-like, gelatinous sheath, which were not observed in *M. submersum*. In addition, *M. submersum* differs from *M. schulzeri* (CBS 325.74) in having smaller conidia (4–5 × 1–2 μm vs. (6–)9–10(–12) × 3–4 µm). Based on the morphology and molecular data, we recognize *M. submersum* as a new species of *Myrmecridium* in this study.

*Neomyrmecridium* Crous

*Notes*: *Neomyrmecridium* was introduced by Crous et al. [81] with *N. septatum* as the type species. The genus is characterized by solitary, unbranched conidiophores, polyblastic, denticulate conidiogenous cells, and fusoid-ellipsoid, septate conidia with the upper two-thirds encased in a mucoid sheath [81,95]. Based on morphological and phylogenetic analyses, nine species are currently recognized in *Neomyrmecridium* (Index Fungorum 2024). Species in this genus have been reported from freshwater and terrestrial habitats in China, Ecuador, Thailand, and Germany [45,81,91,95,96,97].

**Figure 12:**
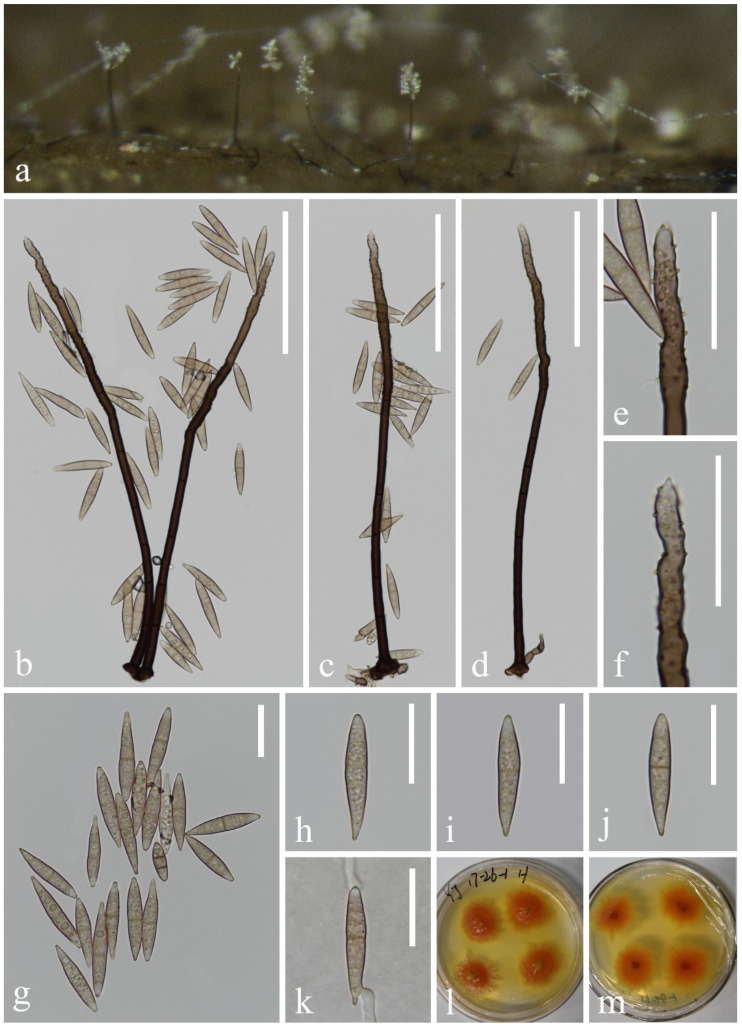
*Neomyrmecridium fusiforme* (KUN-HKAS 132122, holotype): (**a**) colonies on submerged decaying wood, (**b**–**d**) conidiophores and conidia, (**e**,**f**) conidiogenous cells, (**g**–**j**) conidia, (**k**) germinating conidium, and (**l**,**m**) culture on PDA from surface and reverse. Scale bars: (**b**–**d**) 80 µm, (**e**,**f**) 30 µm, and (**g**–**k**) 10 µm.

*Neomyrmecridium fusiforme* L. Zhang & Z.L. Luo sp. nov., Figure 12

Fungal Names number: FN 572054

Etymology: Referring to the fusiform conidia of this fungus.

Holotype: KUN-HKAS 132122

*Saprobic* on submerged decayed wood in freshwater streams. **Sexual morph**: Undetermined. **Asexual morph**: Hyphomycetous. *Colonies* on natural substrate effuse, greyish white, velvety. *Mycelium* immersed, composed of brown, branched, septate hyphae. *Conidiophores* 214–258 × 4–5 μm ($\bar{x}$ = 236 × 5 μm, n = 15), macronematous, mononematous, solitary, erect, unbranched, straight or slightly flexuous, subcylindrical, smooth-walled, septate, dark brown, paler towards apex. *Conidiogenous cells* polyblastic, terminal, integrated, subcylindrical, pale brown, with several denticles at the apex. *Conidia* 29–34 × 4–6 μm ($\bar{x}$ = 32 × 5 μm, n = 35), solitary, fusiform, navicular to tapering and pointed at both ends, pale brown, paler at both ends, 0–3-septate, mostly uniseptate, guttulate, smooth-walled.

*Culture characteristics*: Conidia germinating on PDA within 24 h. Germ tubes are produced from one or both ends. Colonies on PDA reaching 32 mm in diameter after 2 weeks at 25 °C in natural light, with dense mycelium on the surface, producing yellow pigment, bright orange at the center, pale yellow on the irregular edge.

*Material examined*: CHINA, Yunnan Province, on submerged decaying wood in a freshwater stream in the Yuanjiang River basin, 23°48′12″ N, 101°47′21″ E, 22 February 2022, Hong-Wei Shen, S-3712 (KUN-HKAS 132122, holotype), ex-type culture, CGMCC 3.27412 = KUNCC 23–17153.

*Notes*: Phylogenetically, *Neomyrmecridium fusiforme* forms a distinct lineage basal to four *Neomyrmecridium* species, namely, *N. aquaticum* (MFLUCC 15–0366 and MFLUCC 18–1489), *N. guizhouense* (GZCC 20–0008), *N. naviculare* (GZCC 20–0484 and MFLUCC 19–0303), and *N. septatum* (CBS 145073) with 99% ML and 1.00 PP support (Figure 10). *N. fusiforme* resembles *N. aquaticum*, *N. guizhouense*, *N. naviculare*, and *N. septatum* in having erect, unbranched, septate, brown conidiophores; integrated, terminal conidiogenous cells; and septate conidia. However, *N. fusiforme* can be distinguished from the four species by the size of conidiophores and conidia, as well as the number of septa (Table 1). Based on the morphology and molecular data, we recognize *N. fusiforme* as a new species of *Neomyrmecridium*.

*Magnaporthales* Thongk, Vijaykr & K.D. Hyde
*Pseudohalonectriaceae* Hongsanan & K.D. Hyde

*Notes*: *Pseudohalonectriaceae* was introduced by Hongsanan et al. [98] with *Pseudohalonectria* as the type, based on the molecular clock analysis of LSU, SSU, *tef*1-α, and *rpb*2. *Pseudohalonectriaceae* species are saprobes on submerged rotten wood or twigs [97,98]. Some *Pseudohalonectria* species can produce azaphilone compounds that have antagonistic activity against several pests, weeds, nematodes, bacteria, and fungi [99,100,101,102,103].

*Pseudohalonectria* Minoura & T. Muroi

*Notes*: *Pseudohalonectria* was introduced by Minoura and Muroi [104] to accommodate *P. lignicola*. The genus is characterized by immersed to semi-immersed, bright yellow to brown ascomata with a long neck, unitunicate, cylindrical, clavate, or fusiform asci with a non-amyloid, thimble-shaped, refractive apical ring, and cylindrical, fusiform, hyaline to slightly pigmented, septate ascospores [97,105,106]. A phialidic asexual morph with hyaline, allantoid, aseptate conidia was described for *P. aomoriensis* and *P. phialidica* [105,107]. Huhndorf et al. [108] later transferred *P. phialidica* to *Ceratosphaeria* based on morphological and phylogenetic analyses. Currently, 15 species are accepted in *Pseudohalonectria* (Index Fungorum 2024). *Pseudohalonectria* species are commonly reported from submerged wood in freshwater habitats [105,109,110,111] or a few are in terrestrial habitats [112]. Only *P. halophila* has been recorded from a marine habitat [113].

*Pseudohalonectria lutea* Shearer, Can. J. Bot. 67: 1950 (1989), Figure 13

**Figure 13:**
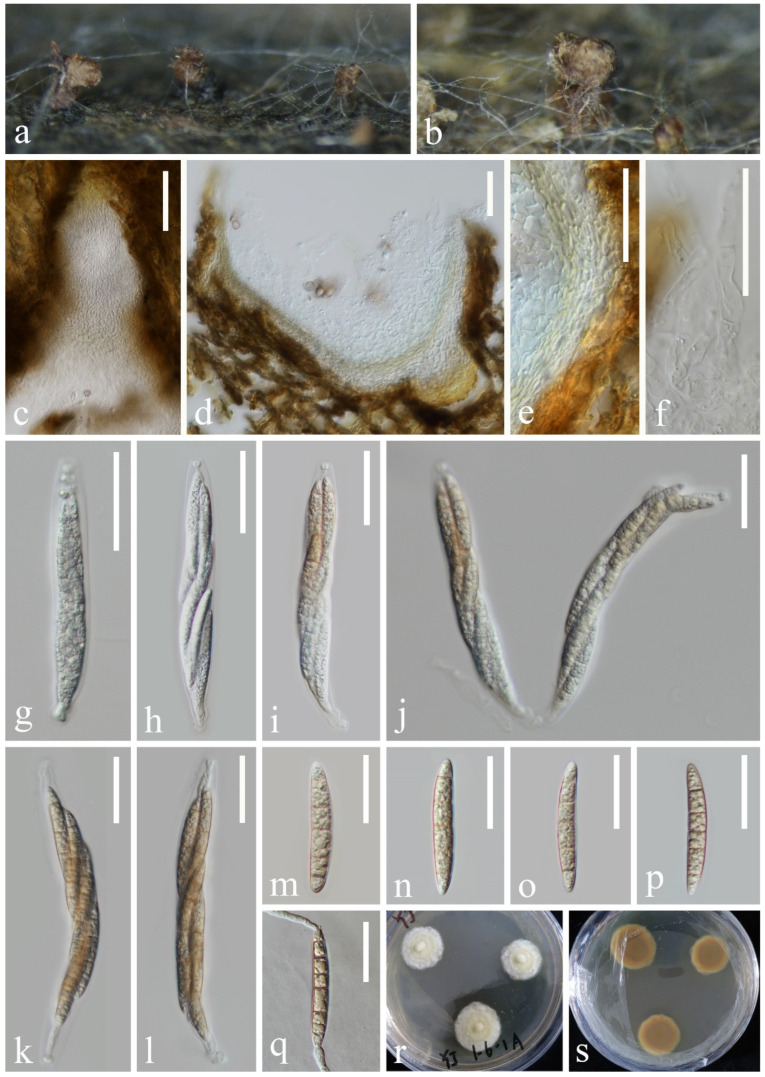
*Pseudohalonectria lutea* (KUN-HKAS 132118): (**a**,**b**) appearance of ascomata on the host, (**c**) ostiole, (**d**) sections of ascomata, (**e**) section of peridium, (**f**) paraphyses, (**g**–**l**) asci, (**m**–**p**) ascospores, (**q**) germinated ascospore, and (**r**,**s**) colony on PDA from above and below. Scale bar: (**c**) 80 µm, (**d**–**l**) 40 µm, and (**m**–**q**) = 30 µm.

Index Fungorum number: IF 136213

*Saprobic* on submerged decayed wood in freshwater stream. **Asexual morph**: Undetermined. **Sexual morph**: Conspicuous as black or brown necks on the host surface and masses of brown ascospores on the apex of necks. *Ascomata* 220–460 × 100–370 μm, scattered or aggregated, immersed, subglobose to obpyriform, yellowish brown becoming dark brown, ostiolate. *Ostiolar* 72–199 µm long, central, papillate, rounded, brown. *Peridium* 12–25 µm thick, outer layer yellowish brown, undifferentiated from plant tissue, inner layer consisting of pale brown to hyaline, elongated, thin-walled cells of textura angularis or textura prismatic. *Paraphyses* septate, hyaline, unbranched, guttulate, tapering towards the apex, 6–8 μm wide near the base, constricted at the septa. *Asci* 134–185 × 15–18 µm ($\bar{x}$ = 160 × 17 µm, n = 30), fusiform, straight or slightly curved, truncate at the apex, 8-spored, with a non-amyloid, thimble-shaped apical ring. *Ascospores* 50–57 × 7–9 µm ($\bar{x}$ = 54 × 8 µm, n = 30), overlapping uniseriate to biseriate, cylindrical-fusiform, straight or slightly curved, hyaline when young, pale brown when mature, 5-septate, smooth-walled, guttulate, thin-walled.

*Culture characteristics*: Ascospores germinating on PDA medium within 24 h. Germ tubes are produced from both ends. Colonies on MEA medium are slow-growing, reaching 10–15 mm in diameter after 1 month at 25 °C in natural light, circular, with regular margins, pale yellowish-green in the center and white at the margin; in reverse, brown in the center and pale brown at the margin.

*Material examined*: China, Yunnan Province, on submerged decaying wood in a freshwater stream in the Yuanjiang River basin, 25°28′05″ N, 100°09′39″ E, 19 February 2022, Hong-Wei Shen, S-3646 (KUN-HKAS 132118), living cultures, KUNCC 24–17896.

**Figure 14:**
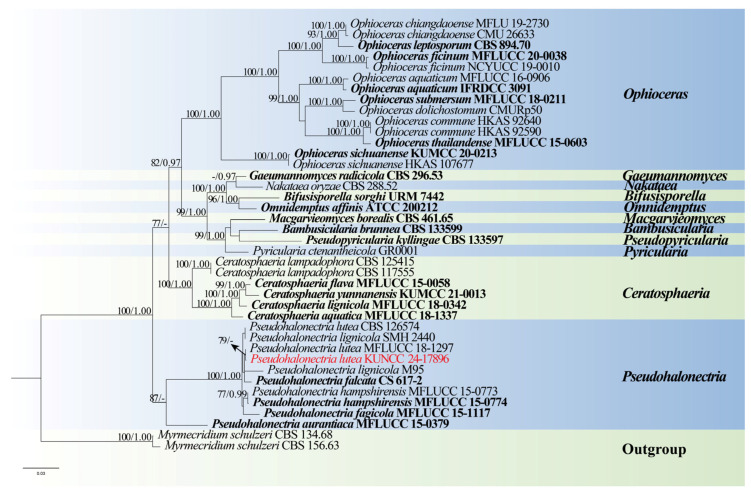
RAxML tree based on analysis of combined ITS, LSU, SSU, and *tef*1-α dataset. The combined analyses include 40 strains with 3376 characters, including gaps (ITS: 537 bp, LSU: 878 bp, SSU: 1036 bp, and *tef*1-α: 925 bp). The tree is rooted to *Myrmecridium schulzeri* (CBS 134.68 and CBS 156.63). Tree topology of the maximum likelihood analysis and Bayesian analysis are similar. The RAxML analysis of the combined dataset yielded a best-scoring tree with a final ML likelihood value of −15046.161199. The matrix had 1026 distinct alignment patterns, with 39.94% undetermined characters or gaps. Estimated base frequencies were as follows: A = 0.243635, C = 0.255589, G = 0.282280, and T = 0.218495; substitution rates AC = 1.177940, AG = 1.732701, AT = 1.671662, CG = 1.330592, CT = 5.639331, and GT = 1.000000; and gamma distribution shape parameter α = 0.187799. Bootstrap values for maximum likelihood (ML) ≥ 75% and clade credibility values of ≥0.95 from Bayesian-inference analysis are labeled on the nodes. The ex-type strains are in bold. The newly obtained sequences are indicated in red.

*Notes*: Phylogenetic analysis showed that our new isolate clustered with *Pseudohalonectria lutea* (MFLUCC 18–1297) (Figure 14). Furthermore, our new isolate exhibits morphological characters identical to *P. lutea* (MFLUCC 18–1297). A comparison of the LSU, *tef*1-α, and SSU sequences between our new strain and the strain MFLUCC 18–1297 reveals only minimal base pair differences. Therefore, based on morphological evidence and phylogenetic affinity, our new strain is identified as *P. lutea*.

## 4. Discussion

Dry-hot valley is one of the areas with an extremely fragile ecological environment. The diversity, distribution pattern, and driving factors of lignicolous freshwater fungi in rivers and streams of dry-hot valleys are not well-studied. The Yuanjiang River basin, the typical representative of dry-hot valleys in southwestern China, is selected as the study site in this study. We investigate the lignicolous freshwater fungi in the Yuanjiang River in Yunnan Province; four new species, two new records, and three known species are reported. The results indicate high undiscovered diversity of lignicolous freshwater fungi in the Yuanjiang River. In recent years, most of the *Dictyosporiaceae* species were reported in China and Thailand [16,41,42,44,45,49,50,51]. Among the nine species collected in this study, four belong to *Dictyosporiaceae*, indicating that the Yuanjiang River basin may be rich in *Dictyosporiaceae* species. Conducting diversity surveys in underexplored ecosystems, such as the Yuanjiang River, can not only fill taxonomic gaps but also provide a more reliable foundation for studies on the diversity and evolution of *Dictyosporiaceae* species. This research enriches the knowledge of the biodiversity of the lignicolous freshwater fungi from dry-hot valleys, as well as accumulates basic data for analyzing the community structure and distribution pattern of lignicolous freshwater fungi from dry-hot valleys.

The classification of several early described fungal species is unclear due to the lack of molecular data and insufficient morphological documentation. Additionally, some morphologically similar species exhibit different phylogenetic relationships, further increasing the complexity of classification. For example, *Pseudohalonectria lignicola* [104] and *P. lutea* [105] were originally described based solely on morphology, and the molecular data for both species has only recently become available. The comparison of the existing sequences for *P. lignicola* (SMH 2440) and *P. lutea* (CBS 126574 and MFLUCC 18–1297) reveals minimal differences between them. Furthermore, the morphology of lignicolous freshwater fungi is influenced by environmental pressures, resulting in both convergent evolution (where distantly related species appear similar) and divergent evolution (where closely related species appear different) [114,115]. Distantly related fungi may exhibit similar morphologies in similar environments, while fungi of the same species or with close phylogenetic relationships may show significant morphological differences in extreme environments [116,117]. The two evolutionary pathways present significant challenges for morphological identification. In practical work, some morphological differences may be attributed to variations in the environmental conditions of the collection sites. These environmental factors could influence the external characteristics of fungi, including color, shape, and size, among others. This makes it increasingly challenging to rely solely on morphological features for identifying different species. Therefore, due to differences in the collection sites, *P. lignicola* (SMH 2440) and *P. lutea* (CBS 126574, MFLUCC 18–1297) could potentially be the same species. Thus, both morphological and molecular data should be considered together when identifying species.

## 5. Conclusions

In this study, based on multigene phylogenetic analysis and morphological characterization, nine species of freshwater ascomycetes were identified, including four new species, namely, *Aquadictyospora aquatica*, *Dictyosporium fluminicola*, *Myrmecridium submersum*, and *Neomyrmecridium fusiforme*, plus two new records for China, *Dictyocheirospora aquadulcis* and *Myrmecridium hydei*, and three known species, *Dictyocheirospora rotunda*, *Halobyssothecium aquifusiforme*, and *Pseudohalonectria lutea*. The Yuanjiang River may contain more undiscovered freshwater fungal species, and we will continue to study the lignicolous freshwater fungi in its environment, aiming to further uncover species diversity, ecological functions, and roles in freshwater ecosystems.

## Figures and Tables

**Table 1:** Comparison of conidia characteristics and habitats of *Neomyrmecridium aquaticum*, *N. fusiforme*, *N. guizhouense*, *N. naviculare*, and *N. septatum*.

Species	Conidiophores	Conidiogenous Cells	Conidia	Host	Distribution	References
*Neomyrmecridium aquaticum*	Macronematous, mononematous, erect, unbranched, multi-septate, 211–308 × 5–7 μm	Holoblastic, polyblastic, integrated, terminal	Obovoid, 3-septate, 14–16 × 4–6 μm	On decaying wood in freshwater habitat	China	[97]
*N. fusiforme*	Macronematous, mononematous, solitary, erect, unbranched, 214–258 × 4–5 μm	Polyblastic, terminal, integrated, subcylindrical	Fusiform, navicular to tapering and pointed at both ends, (0–)1–(3–)septate, 29–34 × 4–6 μm	On decaying wood in freshwater habitat	China	This study
*N. guizhouense*	Macronematous, mononematous, solitary, erect, unbranched, 75–140 × 2–4.5 μm	Polyblastic, terminal, integrated, subcylindrical, 2.2–4.3 μm	Fusoid-ellipsoid, (2–)3-septate, 8.9–12.7 × 2.8–4.8 μm	On decaying wood in freshwater habitat	China	[95]
*N. naviculare*	Macronematous, mononematous, erect, unbranched, 100–200 × 4–5.6 µm	Polyblastic, integrated, terminal, sympodial, denticulate	Navicular to fusiform, tapering to a hilum towards the base, (1–)3–septate, 16–24 × 5.5–7.5 µm, with a thin mucilaginous sheath	On decaying submerged wood in freshwater habitats	China	[45]
*N. septatum*	Solitary, erect, straight, unbranched, 1–4-septate, 40–70 × 4–5 µm	Polyblastic, terminal, integrated, denticles, 30–40 × 4–5 µm	Fusoid-ellipsoid, 1–3-septate, 12–20 × 3.5–5 µm, mucoid sheath	On leaves of unidentified vine	Thailand	[81]

## Data Availability

The data presented in this study are openly available in the Index Fungorum (http://www.indexfungorum.org/names/names.asp, accessed on 21 October 2024) and GenBank (https://www.ncbi.nlm.nih.gov/nuccore, accessed on 21 October 2024).

## Supplementary Materials

The following supporting information can be downloaded at: https://www.mdpi.com/article/10.3390/jof10120881/s1, Table S1. Strains/ Voucher used for phylogenetic analysis and their GenBank accession numbers. The ex-type strains were indicated by ^T^ after the Strain/ Voucher number, newly generated species, strains, and sequences are in red.
