# Supplementary material for: Diversity of Lignicolous Freshwater Fungi from Yuanjiang River in Yunnan (China), with the Description of Four New Species
Source: J Fungi (Basel). 2024 Dec 18;10(12):881. doi: 10.3390/jof10120881 (PMC11676937; doi:10.3390/jof10120881)
Supplement: Supplementary file 1 [file jof-10-00881-s001.zip › jof-3311602-supplementary.pdf]

Journal of Fungi

MDPI

Table S1. Strains/ Voucher used for phylogenetic analysis and their GenBank accession numbers. The ex-type strains were indicated by ^{T} after the Strain/ Voucher number, newly generated species, strains, and sequences are in red.

Species	Strain/Voucher	GenBank Accession No.			
		ITS	LSU	SSU	*tef*1-α
Aquadictyospora aquatica	KUNCC 23–17142^{T}	PQ038337	PQ226143	–	PQ227065
Aquadictyospora clematidis	MFLUCC 17–2080^{T}	MT310592	MT214545	–	MT394727
Aquadictyospora fluviatilis	CGMCC 3.27012^{T}	OR831978	OR836027	–	PQ465193
Aquadictyospora lignicola	MFLUCC 17–1318^{T}	MF948621	MF948629	–	MF953164
Aquadictyospora yunnanensis	ZHKUCC 22–0280	OQ544399	OQ544397	–	OQ556796
Aquadictyospora yunnanensis	ZHKUCC 22–0279^{T}	OQ544398	OQ544396	–	OQ556795
Bambusicularia brunnea	CBS 133599^{T}	NR145387	NG058671	–	–
Bifusisporella sorghi	URM 7442^{T}	NR164042	NG067852	–	MK060157
Ceratosphaeria aquatica	MFLUCC 18–1337^{T}	MK828612	MK835812	–	MN194065
Ceratosphaeria flava	MFLUCC 15–0058^{T}	OP377883	OP377969	OP378046	–
Ceratosphaeria lampadophora	CBS 125415	MH863598	MH875074	–	–
Ceratosphaeria lampadophora	CBS 117555	–	AY761084	AY761088	–
Ceratosphaeria lignicola	MFLUCC 18–0342^{T}	MK828613	MK835813	–	MN194066
Ceratosphaeria yunnanensis	KUMCC 21–0013^{T}	–	OL473547	–	OL505597
Cheirosporium triseriale	HMAS 180703^{T}	EU413953	EU413954	–	–
Crassoascoma potentillae	UESTCC 21.0010	OK161237	OK161254	OK161233	OK181165
Crassoascoma potentillae	CGMCC 3.20483^{T}	OK161240	OK161257	OK161236	OK181168
Darksidea alpha	CBS 135650^{T}	NR137619	KP184019	KP184049	KP184166
Darksidea gamma	CBS 135634^{T}	NR137587	KP184031	KP184073	KP184188
Darksidea zeta	CBS 135640^{T}	NR137958	KP184013	KP184071	KP184191
Dendryphiella eucalyptorum	CBS 137987^{T}	KJ869139	KJ869196	–	–
Dendryphiella fasciculata	MFLUCC 17–1074^{T}	MF399213	MF399214	–	–
Dendryphiella paravinosa	CBS 141286^{T}	KX228257	KX228309	–	–
Dictyocheirospora acaciae	SDBR CMU454	OP965332	OP965372	–	OQ000838
Dictyocheirospora acaciae	SDBR CMU455	OP965333	OP965373	–	OQ000839
Dictyocheirospora aquadulcis	MFLUCC 17–2571^{T}	MK634545	MK634542	–	–
Dictyocheirospora aquadulcis	MFLUCC 22–0095	OP526634	OP526644	–	OP542236
Dictyocheirospora aquadulcis	KUNCC 23–17204	PQ037173	PQ226137	–	PQ227063
Dictyocheirospora aquadulcis	KUNCC 23–17067	PQ037172	PQ226136	–	PQ227062
Dictyocheirospora aquatica	KUMCC 15–0305^{T}	KY320508	KY320513	–	–
Dictyocheirospora bannica	KH 332^{T}	LC014543	AB807513	–	AB808489
Dictyocheirospora bannica	MFLUCC 16–0874	MH381765	–	–	–
Dictyocheirospora cheirospora	KUMCC 17–0035^{T}	MF177035	MF177036	–	–
Dictyocheirospora chiangmaiensis	MFLUCC 22–0097^{T}	OP526630	OP526640_	–	OP542232
Dictyocheirospora clematidis	MFLUCC 17–2089^{T}	MT310593	MT214546	–	MT394728
Dictyocheirospora garethjonesii	MFLUCC 16–0909^{T}	KY320509	KY320514	–	–
Dictyocheirospora garethjonesii	DLUCC 0848	MF948623	MF948631	–	MF953166
Dictyocheirospora gigantica	BCC 11346	DQ018095	–	–	–
Dictyocheirospora heptaspora	CBS 396.59	DQ018090	–	–	–
Dictyocheirospora indica	MFLUCC 15–0056	MH381763	MH381772	–	MH388817
Dictyocheirospora lithocarpi	MFLUCC 17–2537^{T}	MK347781	MK347999	–	–
Dictyocheirospora metroxylonis	MFLUCC 15–0028a^{T}	MH742321	MH742313	–	–
Dictyocheirospora metroxylonis	MFLUCC 15–0028b	MH742322	MH742314	–	MH764303
Dictyocheirospora multiappendiculata	KUNCC 22–10734^{T}	OP526632	OP526642	–	OP542234

Species	Strain/Voucher	GenBank Accession No.			
		ITS	LSU	SSU	*tef*1-α
Dictyocheirospora multiappendiculata	KUNCC 22–10736	OP526633	OP526643	–	OP542235
Dictyocheirospora nabanheensis	KUMCC 16–0152	MH388340	MH376712	–	MH388375
Dictyocheirospora pandanicola	MFLUCC 16–0365[T]	MH388341	MH376713	–	MH388376
Dictyocheirospora pseudomusae	yone 234[T]	LC014550	AB807520	–	AB808496
Dictyocheirospora rotunda	MFLUCC 14–0293[T]	KU179099	KU179100	–	–
Dictyocheirospora rotunda	MFLUCC 17–0222	MH381764	MH381773	–	MH388818
Dictyocheirospora rotunda	KUNCC 23–17140	PQ220114	PQ226139	–	–
Dictyocheirospora rotunda	KUNCC 23–17124	PQ220115	PQ226140	–	PQ227064
Dictyocheirospora suae	KUNCC 22–12424[T]	OP526631	OP526641	–	OP542233
Dictyocheirospora submers	ZHKUCC 24–0001[T]	PP326193	PP326216	–	PP333113
Dictyocheirospora subramanianii	BCC 3503	DQ018094	–	–	–
Dictyocheirospora taiwanense	MFLUCC 17–2654[T]	MK495821	MK495820	–	–
Dictyocheirospora thailandica	MFLUCC 18–0987[T]	MT627734	MN913743	–	–
Dictyocheirospora vinaya	MFLUCC 14–0294[T]	KU179102	KU179103	–	–
Dictyocheirospora xishuangbannaensis	KUMCC 17–0181[T]	MH388342	MH376714	–	MH388377
Dictyosporium alatum	ATCC 34953[T]	NR077171	DQ018101	–	–
Dictyosporium appendiculatum	KUNCC 17–0311[T]	MH388343	MH376715	–	–
Dictyosporium aquaticum	MF 1318[T]	KM610236	–	–	–
Dictyosporium bulbosum	yone 221[T]	LC014544	AB807511	–	AB808487
Dictyosporium cycadicola	HKAS 134909[T]	PP740382	PP740388	–	PP776570
Dictyosporium digitatum	KH 401	LC014545	AB807515	–	AB808491
Dictyosporium digitatum	yone 280	LC014547	AB807512	–	AB808488
Dictyosporium duliujiangense	GZCC 19–0426[T]	OQ842725	MW133815	–	OQ850746
Dictyosporium elegans	NBRC 32502[T]	DQ018087	DQ018100	–	–
Dictyosporium fluminicola	CGMCC 3.27408[T]	PQ038338	PQ226144	–	PQ227066
Dictyosporium guangdongense	ZHKUCC 24–0002[T]	PP326190	PP326213	–	–
Dictyosporium guttulatum	MFLUCC 16–0258[T]	MH388345	MH376717	–	MH388379
Dictyosporium hongkongensis	KMUCC 17–0268[T]	MH388346	MH376718	–	MH388380
Dictyosporium hughesii	KT 1847[T]	LC014548	AB807517	–	AB808493
Dictyosporium karsti	MFLU 18–2282[T]	OR225025	OP099521	–	OR140390
Dictyosporium krabiense	MFLU 16–1890[T]	–	MH376719	–	MH388381
Dictyosporium licualae	GZCC 21–0270[T]	PP594915	–	–	PP740420
Dictyosporium marinum	GJ 357[T]	–	MN017841	–	–
Dictyosporium meiosporum	MFLUCC 10–0131[T]	KP710944	KP710945	–	–
Dictyosporium muriformis	GZCC 20–0006	MT002304	MN897834	–	MT023011
Dictyosporium nigroapice	BCC 3555	DQ018085	–	–	–
Dictyosporium nigroapice	MFLUCC 17–2053	MH381768	MH381777	–	MH388821
Dictyosporium olivaceosporum	KH 375[T]	LC014542	AB807514	–	AB808490
Dictyosporium palmae	CBS-H 22129[T]	–	KX555648	–	–
Dictyosporium pandanicola	MFLU 16–1886[T]	MH388347	MH376720	–	MH388382
Dictyosporium sexualis	MFLUCC 10–0127[T]	KU179105	KU179106	–	–
Dictyosporium sp.	MFLUCC 15–0629	MH381766	MH381775	–	MH388819
Dictyosporium stellatum	CCFC 241241[T]	NR154608	JF951177	–	–
Dictyosporium strelitziae	CBS 123359[T]	NR156216	FJ839653	–	–
Dictyosporium tetrasporum	KT 2865	LC014551	AB807519	–	AB808495
Dictyosporium thailandicum	MFLUCC 13–0773[T]	KP716706	KP716707	–	–
Dictyosporium tratense	MFLUCC 17–2052[T]	MH381767	MH381776	–	MH388820
Dictyosporium tubulatum	MFLUCC 15–0631[T]	MH381769	MH381778	–	MH388822
Dictyosporium tubulatum	MFLUCC 17–2056	MH381770	MH381779	–	–

Dictyosporium variabilisporum	ZHKUCC 24–0003	PP326192	PP326215	–	PP333112
Dictyosporium wuyiense	CGMCC 3.18703^{T}	KY072977	–	–	–
Dictyosporium zhejiangense	MW-2009a^{T}	FJ456893	–	–	–
Digitodesmium bambusicola	CBS 110279	DQ018091	DQ018103	–	–
Digitodesmium chiangmaiense	KUN-HKAS 102163^{T}	–	MK571766	–	–
Digitodesmium polybrachiatum	COAD 3174^{T}	MW879318	MW879316	–	–
Digitodesmium polybrachiatum	COAD 3175	MW879319	MW879317	–	–
Gaeumannomyces radicicola	CBS 296.53^{T}	–	NG058089	KM009218	KM009206
Gregarithecium curvisporum	KT 922^{T}	AB809644	AB807547	–	–
Gregarithecium curvisporum	MFLUCC 13–0853	KX364281	KX364282	–	–
Halobyssothecium aquifusiforme	GZCC 20–0481^{T}	OP377825	OP377925	OP378010	OP473005
Halobyssothecium aquifusiforme	KUNCC 23-17064	PQ220116	PQ226141	PQ226133	PQ227067
Halobyssothecium aquifusiforme	MFLUCC 19–0305	OP377829	OP377929	OP378014	OP473008
Halobyssothecium bambusicola	MFLUCC 20–0226^{T}	MN833419	MT068489	MT068494	MT477868
Halobyssothecium cangshanense	DLUCC 0143^{T}	–	KU991149	KU991150	–
Halobyssothecium caohaiense	GZCC 19–0482^{T}	OP377841	MW133831	MW134611	OP473019
Halobyssothecium carbonneanum	CBS 144076^{T}	MH062991	MH069699	–	–
Halobyssothecium estuariae	MFLUCC 19–0386^{T}	MN598890	MN598871	MN598868	MN597050
Halobyssothecium estuariae	MFLUCC 19–0387^{T}	MN598891	MN598872	MN598869	MN597051
Halobyssothecium kunmingense	KUMCC 19–0101^{T}	MT627715	MN913732	MT864313	MT954408
Halobyssothecium obiones	20AV2566	KX263862	–	–	–
Halobyssothecium obiones	27AV2385	KX263864	–	–	–

**Species**	**Strain/Voucher**	**GenBank Accession No.**			
		**ITS**	**LSU**	**SSU**	***tef*1-α**
Halobyssothecium obiones	MFLUCC 15–0381^{T}	MH377060	MH376744	MH376745	MH376746
Halobyssothecium phragmitis	MFLUCC 20–0225	MT232437	MT068488	MT068493	MT477867
Halobyssothecium phragmitis	HKAS 127181	OR506177	OR506189	OR506192	OR513794
Halobyssothecium phragmitis	MFLUCC 20–0223^{T}	MT232435	MT068486	MT068491	MT477865
Halobyssothecium sichuanense	CN12^{T}	ON124829	ON124913	–	–
Halobyssothecium thailandica	MFLUCC 21–0062^{T}	MZ429434	MZ433248	MZ429435	–
Halobyssothecium unicellulare	MD129	–	KX505375	KX505373	–
Halobyssothecium unicellulare	KUNCC 22–12413	OR335290	OR335347	OR335330	–
Halobyssothecium unicellulare	MD6004^{T}	–	KX505376	KX505374	–
Halobyssothecium versicolor	MFLUCC 20–0222^{T}	MT232434	MT068485	MW346047	MT477864
Halobyssothecium voraginesporum	CBS H-22560^{T}	–	KX499520	KX499519	–
Immotthia bambusae	KUN-HKAS 112012AI^{T}	MW489455	MW489450	–	MW504646
Immotthia bambusae	KUN-HKAS 112012AII^{T}	MW489456	MW489451	–	MW504647
Immotthia bambusae	KUN-HKAS 112012B^{T}	MW489457	MW489452	–	–
Jalapriya inflata	NOU 3855	JQ267362	JQ267363	–	–
Jalapriya pulchra	MFLUCC 15–0348^{T}	KU179108	KU179109	–	–
Jalapriya pulchra	MFLUCC 17–1683	MF948628	MF948636	–	MF953171
Jalapriya toruloides	CBS 209.65	DQ018093	DQ018104	–	–
Lanspora cylindrospora	NFCCI 4665	MN168889	MN168891	–	–
Lanspora cylindrospora	NFCCI 4427^{T}	MN168890	MN168892	–	–
Lentithecium clioninum	KT 1149A^{T}	LC014566	AB807540	AB797250	AB808515
Lentithecium clioninum	KT 1220	LC014567	AB807541	AB797251	AB808516
Lentithecium fluviatile	CBS 122367	–	FJ795451	FJ795493	GU349074
Lentithecium fluviatile	CBS 123090	–	FJ795450	FJ795492	–
Lentithecium pseudoclioninum	KT 1113^{T}	AB809632	AB807544	AB797254	AB808520
Lentithecium pseudoclioninum	GZCC 19–0483	OM692194	MW133832	MW134612	–
Lentithecium pseudoclioninum	KUNCC 22–12414	OR335291	OR335348	OR335331	–

Lentithecium pseudoclioninum	KUNCC 22–12415	OR335292	OR335349	OR335332	–
Lentithecium yunnanensis	KUNCC 22–10776^{T}	ON227126	ON227127	ON227123	ON228074
Lentithecium yunnanensis	KUNCC 22–10777	ON227125	ON227124	ON227122	ON228075
Lentithecium yunnanensis	KUNCC 22–12420	OR335293	OR335350	OR335333	OR367664
Macgarvieomyces borealis	CBS 461.65^{T}	NR145384	NG058088	NG061049	KM009198
Myrmecridium banksiae	CBS 132536^{T}	NR111762	NG042684	–	–
Myrmecridium dactylidis	CBS 148281^{T}	OK664729	OK663768	–	–
Myrmecridium flexuosum	CBS 398.76^{T}	NR146238	EU041825	–	–
Myrmecridium fluviae	CNUFC YR61–1^{T}	NR164555	KX839677	–	–
Myrmecridium hiemale	CBS 141017^{T}	NR155370	KU302612	–	–
Myrmecridium hydei	KUNCC 23–17205	PQ037174	–	–	–
Myrmecridium hydei	KUNCC 23–17206	PQ037175	PQ226139	–	–
Myrmecridium hydei	MFLUCC 23–0217^{T}	OR500543	OR500545	–	–
Myrmecridium hydei	MFLUCC 23–0218	OR500544	OR500546	–	–
Myrmecridium iridis	CBS 139917^{T}	NR156299	NG058171	–	–
Myrmecridium junci	CBS 148274^{T}	OK664725	OK663764	–	–
Myrmecridium juncicola	CBS 148316^{T}	OK664731	OK663770	–	–
Myrmecridium juncigenum	CBS 148268^{T}	OK664735	OK663774	–	–
Myrmecridium mexiae	BRIP 69701^{T}	OM417274	OM333588	–	–
Myrmecridium montsegurinum	PRM 934684^{T}	KT991674	KT991664	–	–
Myrmecridium normannianum	CBS 149439^{T}	OP675888	OP681177	–	–
Myrmecridium obovoideum	HGUP 0314^{T}	KC136140	KC136139	–	–
Myrmecridium phragmiticola	CPC 36367^{T}	NR170826	NG074444	–	–
Myrmecridium phragmitigenum	CBS 148945^{T}	ON603769	ON603789	–	–
Myrmecridium phragmitis	CBS 131311^{T}	NR137782	NG057948	–	–
Myrmecridium pulvericola	DAOM 250405^{T}	NR155382	KU309313	–	–
Myrmecridium sambuci	CBS 148444^{T}	OK664707	OK663746	–	–
Myrmecridium schulzeri	CBS 134.68	EU041770	EU041827	–	–
Myrmecridium schulzeri	CBS 156.63	EU041771	EU041828	–	–
Myrmecridium schulzeri	CBS 325.74^{T}	EU041775	EU041832	–	–
Myrmecridium schulzeri	CBS 134.68	EU041770	EU041827	–	–

**Species**	**Strain/Voucher**	**GenBank Accession No.**			
		**ITS**	**LSU**	**SSU**	***tef*1-α**
Myrmecridium spartii	CBS 140006^{T}	NR155376	KR611902	–	–
Myrmecridium splendidum	GZCC 19–0549^{T}	MW133875	OP377931	–	–
Myrmecridium submersum	CGMCC 3.27410^{T}	PQ038339	PQ226145	–	–
Myrmecridium thailandicum	CBS 136551^{T}	NR137605	KF777222	–	–
Myrmecridium yunnanense	GZAAS 23–0586^{T}	OR438389	OR438853	–	–
Myrmecridium yunnanense	GZCC 23–0580	OR438390	OR438854	–	–
Nakataea oryzae	CBS 288.52	MH857040	MH868571	–	–
Neodendryphiella michoacanensis	FMR 16098^{T}	LT906660	LT906658	–	–
Neodendryphiella tarraconensis	FMR 16234^{T}	LT906659	LT906656	–	–
Neodendryphiella tarraconensis	GZCC 20–0002	MN999922	MN999927	–	–
Neomyrmecridium aquaticum	MFLUCC 15–0366^{T}	MK828657	MK849804	–	–
Neomyrmecridium aquaticum	MFLUCC 18–1489	MK828656	MK849803	–	–
Neomyrmecridium asiaticum	CBS 145080^{T}	MK047444	MK047494	–	–
Neomyrmecridium asymmetricum	CCMCIBE H304^{T}	MN014057	MN014055	–	–
Neomyrmecridium asymmetricum	CCMCIBE H304 A	MN014058	MN014056	–	–
Neomyrmecridium fusiforme	CGMCC 3.27412^{T}	PQ038340	PQ226146	–	–
Neomyrmecridium gaoligongense	KUNCC 10794^{T}	OP326185	OP326197	–	–
Neomyrmecridium gaoligongense	KUNCC 10795	OP326186	OP326198	–	–

Neomyrmecridium guizhouense	GZCC 20–0008^{T}	MT002305	MT002307	–	–
Neomyrmecridium luguense	KUNCC 10796^{T}	OP326187	OP326199	–	–
Neomyrmecridium luguense	KUNCC 10797	OP326188	OP326200	–	–
Neomyrmecridium naviculare	GZCC 20–0484^{T}	OP377827	OP377927	–	–
Neomyrmecridium naviculare	MFLUCC 19–0303	OP377828	OP377928	–	–
Neomyrmecridium septatum	CBS 145073^{T}	MK047442	MK047492	–	–
Neomyrmecridium sorbicola	CBS 143433^{T}	MH107901	MH107948	–	–
Omnidemptus affinis	ATCC 200212^{T}	NR154292	NG059478	NG061138	JX134700
Ophioceras aquaticum	IFRDCC 3091^{T}	JQ797440	JQ797433	JQ797435	–
Ophioceras aquaticum	MFLUCC 16–0906	MK828611	MK835810	–	–
Ophioceras chiangdaoense	CMU 26633	–	NG066356	NG065556	–
Ophioceras chiangdaoense	MFLU 19–2730	–	MW114438	MW114307	–
Ophioceras commune	HKAS 92590	MK828610	MK835809	MK834732	MN194062
Ophioceras commune	HKAS 92640	MH795813	MH795818	–	MH801192
Ophioceras dolichostomum	CMURp50	–	DQ341504	DQ341482	–
Ophioceras ficinum	MFLUCC 20–0038^{T}	–	MW114436	–	–
Ophioceras ficinum	NCYUCC 19–0010	–	MW114437	–	–
Ophioceras leptosporum	CBS 894.70^{T}	NR111768	NG057959	JX134664	JX134704
Ophioceras sichuanense	HKAS 107677	MW057782	MW057779	MW057847	–
Ophioceras sichuanense	KUMCC 20–0213^{T}	MT995045	MT995046	MT995047	–
Ophioceras submersum	MFLUCC 18–0211^{T}	–	MK835811	–	MN194064
Ophioceras thailandense	MFLUCC 15–0603^{T}	OP377882	OP377968	OP378045	–
Paralentithecium aquaticum	CBS 123099^{T}	NR160229	GU301823	GU296156	GU349068
Paralentithecium suae	CGMCC 3.24265^{T}	OQ874972	OQ732683	OQ875040	OR367672
Periconia igniaria	CBS 379.86	LC014585	AB807566	–	AB808542
Periconia igniaria	CBS 845.96	LC014586	AB807567	–	AB808543
Pseudocoleophoma bauhiniae	MFLUCC 17–2586^{T}	MK347736	MK347953	–	MK360076
Pseudocoleophoma bauhiniae	MFLUCC 17–2280	MK347735	MK347952	–	MK360075
Pseudocoleophoma calamagrostidis	KT 3284^{T}	LC014592	LC014609	–	LC014614
Pseudocoleophoma flavescen	CBS 178.93	–	GU238075	–	–
Pseudocoleophoma polygonicola	KT 731^{T}	AB809634	AB807546	–	AB808522
Pseudocoleophoma rusci	MFLUCC 16–1444^{T}	MT185549	MT183514	–	–
Pseudocoleophoma typhicola	MFLUCC 16–0123^{T}	KX576655	KX576656	–	–
Pseudocoleophoma zingiberacearum	NCYUCC 19–0052^{T}	MN615939	MN616753	–	MN629281
Pseudocoleophoma zingiberacearum	NCYUCC 19–0053	MN615940	MN616754	–	MN629282
Pseudoconiothyrium broussonetiae	CBS 145036	MK442618	MK442554	–	–
Pseudocyclothyriella clematidis	MFLUCC 17–2177^{T}	MT310596	MT214549	–	MT394730
Pseudocyclothyriella clematidis	MFLUCC 17–2177A^{T}	MT310595	MT214548	–	–
Pseudodictyosporium elegans	CBS 688.93^{T}	DQ018099	DQ018106	–	–
Pseudodictyosporium indicum	CBS 471.95	DQ018097	–	–	–
**Species**	**Strain/Voucher**	**GenBank Accession No.**			
		**ITS**	**LSU**	**SSU**	***tef*1-α**
Pseudodictyosporium thailandica	MFLUCC 16–0029^{T}	KX259520	KX259522	–	KX259526
Pseudodictyosporium wauense	NBRC 30078^{T}	DQ018098	DQ018105	–	–
Pseudodictyosporium wauense	DLUCC 0801	MF948622	MF948630	–	MF953165
Pseudohalonectria aurantiaca	MFLUCC 15–0379^{T}	OP377881	OP377967	OP378044	–
Pseudohalonectria fagicola	MFLUCC 15–1117^{T}	–	KX426219	KX426223	KX426226
Pseudohalonectria falcata	CS 617–2^{T}	–	–	AF050477	–
Pseudohalonectria hampshirensis	MFLUCC 15–0774^{T}	–	KX426218	KX426221	KX426224
Pseudohalonectria hampshirensis	MFLUCC 15–0773	–	KX426220	KX426222	KX426225
Pseudohalonectria lignicola	SMH 2440	–	AY346299	–	–

Pseudohalonectria lignicola	M95	JX134679	JX134691	JX134665	JX134705
Pseudohalonectria lutea	KUNCC 24–17896	PQ220117	PQ226142	PQ226134	PQ227068
Pseudohalonectria lutea	MFLUCC 18–1297	–	MK063809	MK063810	MK099812
Pseudohalonectria lutea	CBS 126574	MH864160	MH875622	–	–
Pseudopyricularia kyllingae	CBS 133597^{T}	NR155645	NG058114	–	–
Pyricularia ctenantheicola	GR0001	KM484878	KM484994	–	–
Verrucoccum coppinsii	E00814291^{T}	MT918785	MT918770	–	–
Verrucoccum spribillei	SPO 1154^{T}	MT918781	MT918764	–	–
Vikalpa australiensis	HKUCC 8797^{T}	DQ018092	–	–	–
Vikalpa grandispora	KUNCC 22–12425^{T}	OP526638	OP526648	–	OP542240
Vikalpa sphaericum	CGMCC 3.20682^{T}	OP526639	OP526649	–	OP542241
